# NSPA: characterizing the disease association of multiple genetic interactions at single-subject resolution

**DOI:** 10.1093/bioadv/vbad010

**Published:** 2023-02-07

**Authors:** Zhendong Sha, Yuanzhu Chen, Ting Hu

**Affiliations:** School of Computing, Queen’s University, Kingston, Ontario, Canada K7L 2N8; School of Computing, Queen’s University, Kingston, Ontario, Canada K7L 2N8; School of Computing, Queen’s University, Kingston, Ontario, Canada K7L 2N8

## Abstract

**Motivation:**

The interaction between genetic variables is one of the major barriers to characterizing the genetic architecture of complex traits. To consider epistasis, network science approaches are increasingly being used in research to elucidate the genetic architecture of complex diseases. Network science approaches associate genetic variables’ disease susceptibility to their topological importance in the network. However, this network only represents genetic interactions and does not describe how these interactions attribute to disease association at the subject-scale. We propose the Network-based Subject Portrait Approach (NSPA) and an accompanying feature transformation method to determine the collective risk impact of multiple genetic interactions for each subject.

**Results:**

The feature transformation method converts genetic variants of subjects into new values that capture how genetic variables interact with others to attribute to a subject’s disease association. We apply this approach to synthetic and genetic datasets and learn that (1) the disease association can be captured using multiple disjoint sets of genetic interactions and (2) the feature transformation method based on NSPA improves predictive performance comparing with using the original genetic variables. Our findings confirm the role of genetic interaction in complex disease and provide a novel approach for gene–disease association studies to identify genetic architecture in the context of epistasis.

**Availability and implementation:**

The codes of NSPA are now available in: https://github.com/MIB-Lab/Network-based-Subject-Portrait-Approach

**Contact:**

ting.hu@queensu.ca

**Supplementary information:**

Supplementary data are available at *Bioinformatics Advances* online.

## 1 Introduction

Genome-wide association studies (GWAS) examine the disease association of single-nucleotide polymorphisms (SNPs) (Visscher *et al.*, 2017). The identified associations are used to describe the genetic architecture of complex diseases and to capture individuals with high genetic risk (Crosby *et al.*, 2022; Edwards *et al.*, 2005; Horowitz *et al.*, 2022; Tam *et al.*, 2019). Since the first GWAS was published 17 years ago (Klein *et al.*, 2005), more than 70 000 SNP–disease associations have been identified as of 2018 (Buniello *et al.*, 2019). Typical statistical approaches adopted by GWAS focus on individual variable effects (main effects). These approaches have been later criticized as the resulting SNPs account for only a fraction of complex disease heritability due to the overlooking of gene–gene interactions (epistasis) (Edwards *et al.*, 2005; Tam *et al.*, 2019). Epistasis has been shown to have a larger contribution to the disease association than the individual variable effect (Boyle *et al.*, 2017; Liu *et al.*, 2019). Therefore, considering epistasis when identifying importance genetic variables can provide a better description of the genetic architecture of complex diseases.

Epistasis describes the non-additive interaction between SNPs that leads to increased susceptibility to complex disease (Boone *et al.*, 2007; Fisher, 1919; Phillips, 2008), where the effect of one genetic variable depends on other variables. For example, the *ERAP1* variant rs30187-T increases disease susceptibility only when the HLA-B*27 or HLA-B*40 alleles are present (Cortes *et al.*, 2015). Epistasis can be a major barrier for GWAS since it is difficult to discover. Searching for disease-relevant variable combinations is computationally prohibitive and may generate false discoveries, as a GWAS dataset can include up to multi-millions of features resulting in an even higher number of feature combinations. According to our literature review, the majority of the current studies are conducted based on second-order feature interactions (Carmelo *et al.*, 2018; Hu *et al.*, 2013). As a result, GWAS require advanced computational approaches to be utilized to identify disease-association epistasis (Moore and Williams, 2009).

Statistical and machine learning methods have been widely adopted in GWAS to capture the effects of genetic epistasis. Multifactor dimensionality reduction (MDR) is one of the most widely adopted methods to capture epistasis (Cattaert *et al.*, 2011; Le *et al.*, 2020; Ritchie *et al.*, 2001). MDR is a parameter-free method for determining the disease association associated with combinations of genetic variables by reducing high-dimensional combinations into a single dimension of disease risk. The major barrier to applying MDR in GWAS is the high-computational cost due to its exhaustive search for all variable combinations. Other machine learning methods have also been adopted to detect epistasis. Random Forest (RF) (Breiman, 2001), for example, has been used to find disease-relevant variables using Gini index (Dorani *et al.*, 2018; Moore *et al.*, 2010; Pan *et al.*, 2013). An extension of RF, known as iterative RF, can be used to discover genetic epistasis by training an ensemble of decision trees with weighted features (Basu *et al.*, 2018). Existing statistical and machine learning methods focus on individual variables or interactions but less frequently investigate the collective disease association of multiple interactions.

Network-based approaches have been proposed to attribute the disease association of genetic variables to the structural importance of the corresponding network nodes (Lareau and McKinney, 2015). Network science studies large-scale empirical networks (Bauer-Mehren *et al.*, 2011; Goh *et al.*, 2007; Newman, 2010), with nodes representing entities and edges representing their interactions. In GWAS, an aggregation of pairwise genetic interactions can be represented as a network (Hu *et al.*, 2011), with nodes representing SNPs or genetic variables and edges connecting different nodes representing genetic interactions. This network can serve as a global map of gene–gene interactions, enabling approaches developed in network science to be well utilized to study the genetic architecture of complex diseases. In the literature, the strength of pair-wise interactions has been quantified using information-theoretical measures such as information gain (Davis *et al.*, 2010; Hu *et al.*, 2011; McKinney *et al.*, 2009) or regression coefficients (Carmelo *et al.*, 2018). Centrality metrics such as degree centrality, eigenvector centrality, and PageRank (Kafaie *et al.*, 2019) can be used to identify nodes with significant structural importance in epistasis network. However, a potential limitation of using node centrality to infer the strength of disease association is that the epistasis network is an averaged representation of genetic interactions in a population. This may overlook the subject-specific risk effects of each interaction (Anholt, 2020). We study the impact of interactions on a subject’s disease risk based on the genetic variants carried by the subject.

The method proposed in this study summarizes the collective risk impact of multiple epistatic interactions on each subject through a feature transformation method. The process transforms each genetic variant by considering the actual risk impact of the corresponding variable interactions, enabling the collective risk impact of pairwise feature interactions to be summarized into individual variables. This study first confirms the effectiveness of the proposed method in identifying epistasis through a simulation study. The proposed method is then applied to a colorectal cancer (CRC) GWAS dataset. We construct an epistasis network to identify significant genetic interactions in the population (Hu *et al.*, 2013). After feature transformation, we use a machine learning algorithm to determine the risk impact of the transformed features as well as the genetic architecture of CRC. Our approach improves genetic risk prediction compared with using genetic variants directly from the GWAS data. Thus, the disease association identified by this method is more convincing than the conventional approach.

## 2 Materials and methods

**Fig. 1.**
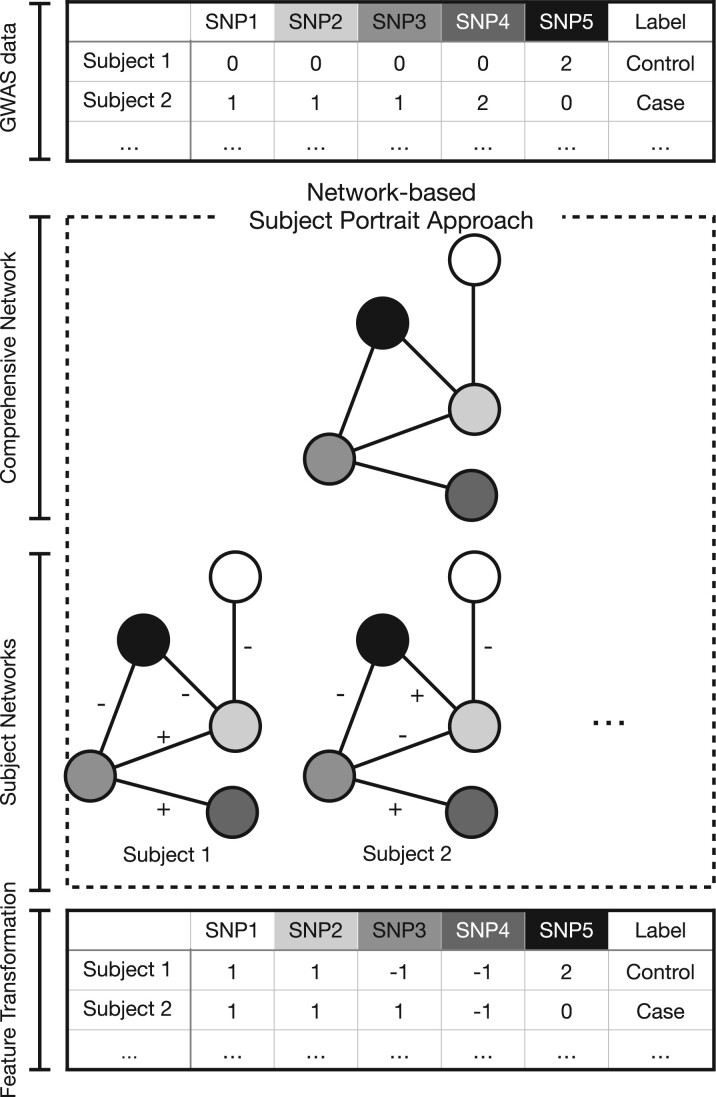
The diagram for the NSPA feature transformation method. This workflow outlines how features are transformed utilizing NSPA (Section 2.2). The content within the dashed box represents NSPA, which includes the comprehensive network (Section 2.3) and the subject-specific subject networks (Section 2.4). The feature transformation method (Section 2.5) is defined on top of the NSPA’s subject networks

The network-based subject portrait approach (NSPA), defined in Section 2.2, utilizes the statistical epistasis network (SEN) (Hu *et al.*, 2011) to further recognize the collective risk impact of multiple genetic interactions through a feature transformation method (see Section 2.5). An illustrative example of the computational procedure is outlined in Figure 1.

### 2.1 CRC GWAS data

CRC begins in the cells of the colon or rectum and may spread to surrounding tissues and other parts of the body. In Canada, more than 26 000 CRC cases were diagnosed in 2020, making CRC the third most commonly diagnosed cancer (Brenner *et al.*, 2020). Early diagnosis of CRC can significantly mitigate the mortality rate of patients, with five-year overall survival statistics ranging from 92% in Stage I to 11% in Stage IV (Crosby *et al.*, 2022; Dienstmann *et al.*, 2017). Colonoscopy, an invasive detection, is a common standard of CRC diagnosis (Lin *et al.*, 2016; Nishihara *et al.*, 2013; Vega *et al.*, 2015). Ideal cancer screening should be minimally invasive or non-invasive (Crosby *et al.*, 2022). Genetic screening has a potential for providing non-invasive early predictions of CRC (Abraham and Inouye, 2015; Ho *et al.*, 2019) as recent genetic-association studies have identified a number of genetic variables associated with an increased risk of CRC (Pearlman *et al.*, 2017).

The GWAS dataset studied in this research is from the CRC Transdisciplinary (CORECT) consortium (Schumacher *et al.*, 2015). Genotyping was conducted using a custom Affymetrix genome-wide platform (the Axiom CORECT Set). A total of 1152 samples (656 CRC cases and 496 controls) were genotyped, with each sample comprised of 265 181 SNPs).

For quality control, a SNP was removed if any of the following conditions hold: (1) the SNP had a call rate lower than 99%, (2) the SNP had a minor allele frequency of lower than 5% or (3) the SNP had a Hardy–Weinberg equilibrium *P*-value lower than 0.0001. A sample was excluded if its missing genotyping rate was greater than 5%, with a discrepancy between sex label and sex chromosome, or had a heterozygosity rate three standard deviations away from the mean. After quality control, a total of 626 cases and 478 controls with 238 196 SNPs was eligible for subsequent analysis.

### 2.2 Network-based subject portrait approach

The NSPA defines two types of networks (Fig. 1). At the population level, we define a comprehensive network of the most significant gene–gene interactions associated with a disease/phenotype. At the subject level, we define a subject network for each subject in the dataset describing the risk contribution of interactions to each subject.

#### 2.2.1 Comprehensive network: SEN

The comprehensive network is defined as the SEN, denoted by $G_{c}=(V,E)$ (Hu *et al.*, 2013, 2011). Each node in *V* corresponds to a genetic variable (or SNP), and an edge in *E* linking a pair of nodes represents a pairwise epistatic interaction. The strength of a pairwise epistatic interaction is quantified using information gain from information theory (Cover and Thomas, 2012). Using an information gain threshold τ, SEN includes only the strong pairwise interactions in *E* with their information gain greater than τ.

Entropy, first proposed by Shannon (1948), quantifies the uncertainty of a random variable. Given a discrete random variable *C*, representing the phenotype outcome of subjects in GWAS {0,1} (control versus case), the entropy of *C* is defined as

(1) $$H(C)=\sum_{c\in C}p(c)\;\text{log}\;\frac{1}{p(c)},$$

where p(c) is the probability estimated using the observed frequency of label c={0,1} of variable *C*. A lower entropy means less uncertainty. Conditional entropy H(C|A) measures the uncertainty of variable *C* given the knowledge of genetic variable *A*:

(2) $$H(C|A)=\sum_{a,c\in A,C}p(a,c)\;\text{log}\;\frac{1}{p(c|a)},$$

where p(a,c) is the probability of having genotype *a* for genetic variable *A* with the random variable *C* labeled as *c* and p(c|a)=p(a,c)/p(a). The margin between H(C) and H(C|A) is the contribution of genetic variable *A* in reducing the uncertainty of *C*, measured as mutual information of *A* and *C*:

(3) I(A;C)=H(C)−H(C|A).

Given the genotypes of a pair of genetic variables *A* and *B*, their gained collective effect on explaining the random variable *C* can be measured using information gain IG(A;B;C):

(4) IG(A;B;C)=I(A,B;C)−I(A;C)−I(B;C).

#### 2.2.2 Subject networks: signed graph based on SEN

The subject network recognizes the actual risk impact of each genetic interaction for each subject with increased or decreased disease susceptibility. In the original GWAS data, each subject is described using a vector of *m* genetic variables denoted by $\langle a_{1},a_{2},\ldots ,a_{m}\rangle \in \Sigma^{m}$, where Σ={0,1,2}. Here, 0 means homozygous reference allele, 1 means heterozygous allele and 2 means homozygous allele. The subject network describes a subject *i* using a signed graph $G_{s(i)}$, which adds a sign to each edge of the comprehensive network for every subject *i*. This allows the comprehensive network $G_{c}$ to depict the disease association of all genetic interactions it contains by considering the subject-specific genotypes.

**Fig. 2.**
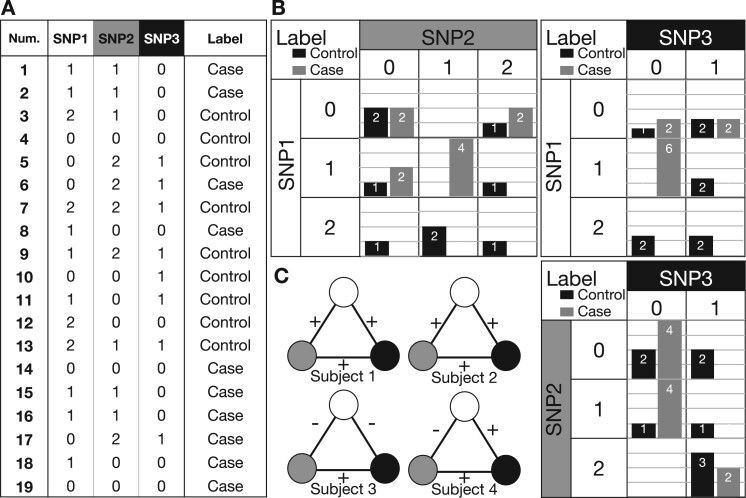
An example of the NSPA subject network. (**A**) A GWAS dataset containing 19 individuals at three SNP sites. (**B**) The panel enumerates all three pairwise contingency tables about the three SNPs. (**C**) The subject networks of the first four subjects in the dataset. The signs of the edges are determined by the distribution of phenotypes in the corresponding contingency table cell based on function σ. As this GWAS dataset is unbalanced, we have appropriately amplified the control samples’ weights by 10/9

The subject network is defined as follows. For each subject *i* in the dataset, we build its subject network $G_{s(i)}(V,E,\chi )$, with the edge sign {+,−} determined by the function χ. Intuitively, a ‘+’ edge indicates a high disease association and a ‘−’ edge means a low disease association. Given an edge e=(u,v) connecting genetic variables *u* and *v*, where the values of *u* and *v* are $a_{u}$ and $a_{v}$, respectively, we define the χ function as

(5) $$\chi (a_{u},a_{v})=\{\begin{array}{cc} ‘+’ & \;\text{if}\;P(a_{u},a_{v},l_{+})\geq P(a_{u},a_{v},l_{-})\\ \;‘-’ & \;\text{otherwise} \end{array},$$

where function *P* gathers the proportion of subjects with variants $a_{u},a_{v}$ and disease status $\left\{l_{+},l_{-}\right\}$ (i.e. ‘case’ or ‘control’) among subjects with the corresponding phenotype outcome (Ritchie *et al.*, 2001). An illustrative example is provided in Figure 2.

### 2.3 Feature transformation method

The feature transformation method considers the actual risk impact of the interactions associated with each genetic variable. For a subject network $G_{s(i)}(V,E,\chi )$, the sign of an edge {+,−} indicates high or low disease association with the corresponding genotype of subject *i*. We extend the definition of node degree centrality to consider the signs of the attached edges. A positive degree $d_{+(i)}(n)$ is defined to consider the number of positive edges and a negative degree $d_{-(i)}(n)$ is defined to consider the number of negative edges. Finally, a delta degree $d_{\Delta (i)}(n)$ is defined as

(6) $$d_{\Delta (i)}(n)=d_{-(i)}(n)-d_{+(i)}(n).$$

We use $d_{\Delta (i)}(n)$ as the transformed value of genetic variable *n* for subject *i*.

### 2.4 Predictive algorithm and feature importance analysis

The transformed features capture not only individual effects of genetic variables but also their combinations. We train predictive models using these transformed features and show that (1) this improves the classification accuracy compared with using the original SNPs directly as features and (2) this allows us to identify importance SNPs associated with the disease through feature importance analysis.

Model training is based on the feature transformation method (Supplementary Fig. S4). In this procedure, the overall dataset is first used to generate a comprehensive network. We then extract genetic variables based on the network and use 5-fold cross-validation (CV) to divide the dataset into five equal folds, using four folds for training and one fold for testing. The observations in the training fold are used to transform the features in both training and testing folds. Finally, we fit a predictive model, such as logistic regression, on the transformed training data to generate a predictive model that considers epistasis. To investigate the role of the feature transformation method, we also use the described procedure to build a predictive model on the data without the feature transformation process.

Logistic regression (Cox, 1958) models the probability of an event using a linear combination of independent variables $\hat{p}=\sigma (\Theta^{T}x)$, where *x* represents the vector of input features, Θ represents the vector of feature weights and logistic function σ is an s-shaped sigmoid function. The learning process will minimize a cost function by adjusting the feature weights vector Θ. We utilize limited-memory Broyden–Fletcher–Goldfarb–Shanno algorithm (Zhu *et al.*, 1997), provided by scikit-learn (Pedregosa *et al.*, 2011), to perform the learning process.

We identify the disease relevance of genetic variables using the weight vector Θ of the logistic regression model. The resampling method (Efron, 1979) replicates multiple logistic regression models (Le *et al.*, 2020). Each replication extracts 80% of observations without replacement from the original dataset. This replication procedure as well as the feature transformation method is repeated 100 times. The feature importance is the averaged weight of the corresponding predictive model across 100 resampling runs.

### 2.5 Functional enrichment using g:Profiler

To interpret the most important SNPs identified, we perform gene functional enrichment analysis using g:Profiler (Raudvere *et al.*, 2019). The tool provides mapping to multiple widely used knowledge resources of Gene Ontology (GO) (Cunningham *et al.*, 2019), including molecular function (GO: MF), biological process (GO: BP) and cellular component (GO: CC). It also includes the most common biological data sources including Kyoto Encyclopedia of Genes and Genomes (Kanehisa *et al.*, 2019), Reactome (Fabregat *et al.*, 2018), WikiPathways (Slenter *et al.*, 2018), Transfac (Matys *et al.*, 2006), miRTarBase (Chou *et al.*, 2018), Human Protein Atlas (Uhlén *et al.*, 2015), CORUM protein complexes (Giurgiu *et al.*, 2019) and Human Phenotype Ontology (Köhler *et al.*, 2019). The statistical significance of functional enrichment terms is quantified using the cumulative hypergeometric test. We consider terms passing the *P*-value lower than 5% as significant.

## 3 Results

Typical GWAS research involves feature selection, predictive model construction and prediction results interpretation to unveil the genetic architecture of complex diseases. We also follow the above procedure with section network investigation for epistasis network describing feature selection, Section 3.3 covering predictive model construction and Section 3.4 explaining the biological interpretation of the model. Prior to applying our approach to the GWAS dataset, we utilize simulation datasets to confirm the capability of the NSPA framework to discover feature interaction.

### 3.1 Synthetic dataset analysis

The synthetic data analysis is conducted on six open datasets from the Penn Machine Learning Benchmarks generated using GAMETES (Romano *et al.*, 2022; Urbanowicz *et al.*, 2012). Among these six datasets, five contain second-order feature interactions and one contains third-order feature interactions.

The goal of the comprehensive network of NSPA is to prioritize epistatic interaction. Therefore, we calculate the value of information gain for all the feature pairs of the simulation dataset and compare the value of the target feature pair with that of the other feature pairs. The results indicate that second-order feature interactions have the highest information gain in all five datasets with pair-wise interaction, suggesting that the comprehensive network can discover second-order feature interactions in the data. However, the information gain of feature interactions involving third-order feature interactions is not significant, suggesting that the comprehensive network formed based on second-order information gain cannot identify feature interactions higher than second order. For complete experiment results, refer to Supplementary Table S1.

### 3.2 Network investigation for epistasis network

NSPA creates a comprehensive network of gene–gene interactions. In this network, a node represents a genetic variable and an edge represents significant pairwise epistatic interaction. The pairwise interaction strength threshold τ regulates the structure of the comprehensive network. The comprehensive network will grow larger as τ decreases, containing more epistatic interactions linking more genetic variables.

**Fig. 3.**
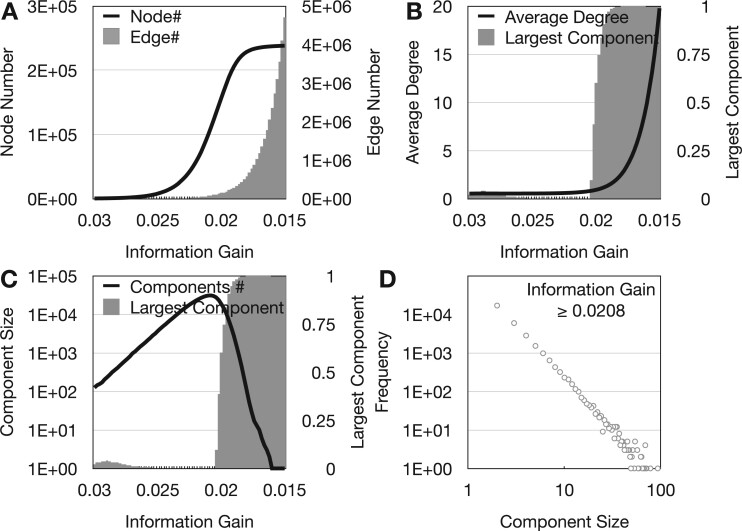
Feature selection based on comprehensive network. (**A**) The evolution of number of edges and nodes in the comprehensive network. (**B**) The evolution of averaged node degree and the size of the largest network component. (**C**) The evolution of network component number. (**D**) The size distribution of network components with edge threshold 0.0208

We investigate various network characteristics to determine the optimal edge cut-off for the comprehensive network, including the number of nodes, number of edges, size of the largest network component and the overall number of network components. As we decrease the edge threshold τ, the evolution of these network characteristics is reported as follows. In Figure 3A, the number of nodes reaches the turning point as the edge threshold decreases to 0.02. The growth then reaches its plateau as the edge threshold reaches 0.016, after which any further 0.002 decreases of edge threshold τ will connect less than 250 new nodes. This result suggests that the network’s growth first concentrates on connecting more nodes and then concentrates on increasing the density of links between nodes. This observation is supported by the evolution of the averaged node degree. As shown in Figure 3B, the average node degree surges after the information gain threshold reaches 0.02. The comprehensive network contains a large number of isolated network components before the largest network component starts to ‘take-over’ the entire network. As shown in Figure 3C, the overall number of network components peaked at the edge threshold 0.0208. This number decreases afterward as the largest network component starts to merge smaller network components, unifying the entire network into one single component.

Feature selection based on threshold τ requires a balance between network connectivity and the strength of edges. If the edge threshold τ is too strict (high), a large number of nodes will not be connected and thus a large amount of relevant information may be lost. If the edge threshold is too low, the network will contain many weak and possibly irrelevant edges, making the relevant information submerged among the irrelevant ones. Given the evolution of various network characteristics, we select τ=0.0208 as the edge threshold. At this point, most nodes have been connected by edges. We consider that the largest network components have the most significant collective disease relevance (Hu *et al.*, 2013). The distribution of node numbers for all network components is shown in Figure 3D. Network components with a node number greater than 60 are selected for subsequent analysis, which includes 15 network components covering 1035 genetic variables. We submit the rsIDs of these genetic variables to g:Profiler, a web server for functional enrichment analysis, using parameters as specified in Supplementary Table S2. The resulting 106 terms are summarized in Supplementary Table S3. Our investigation using EnrichmentMap and AutoAnnotate tools (Reimand *et al.*, 2019) reveals that the results of the biological enrichment analysis cover three major themes, namely signal transduction regulation (*N* = 17), anatomical structure development (*N* = 10) and nervous system development (*N* = 10).

### 3.3 Constructing predictive algorithm based on feature transformation method

Epistasis network does not inform how its interactions contribute to each subject’s genetic risk. For example, the risk impact of an epistatic interaction on a subject can be either a stimulus or a reduction. The proposed NSPA-based feature transformation method (Section 2.5) transforms each genetic variable by considering the risk impact of its interactions with others.

We evaluate the predictive performance of the transformed features by performing CV using a logistic regression algorithm. To determine the role of feature transformation, we also construct predictive models on the original data using logistic regression and RF algorithms. The reason for using RF is that features derived from the comprehensive network may contain feature interactions. The resulting predictive performance suggests substantial improvements compared with the situation when feature transformation is absent. As shown in Table 1, a predictive algorithm with a feature transformation method yields a testing accuracy of 0.752, which surpasses the performance when feature transformation is absent (logistic regression: 0.504 and RF: 0.514).

**Table 1.** The predictive performance of NSPA-based feature transformation (1035 variables)

Conf.	Precision		Recall		F1-score		Acc.
	Case	Ctl	Case	Ctl	Case	Ctl	
Ori_LR	0.563	0.428	0.556	0.435	0.559	0.431	0.504
Ori_RF	0.568	0.437	0.585	0.420	0.576	0.428	0.514
Delta	0.783	0.718	0.763	0.737	0.772	0.727	0.752

*Note*: *Delta* represents building predictive model using logistic regression algorithm on the dataset with feature transformation. *Ori_LR* and *Ori_RF* represent building predictive models using logistic regression and RF algorithms on the dataset without feature transformation.

**Fig. 4.**
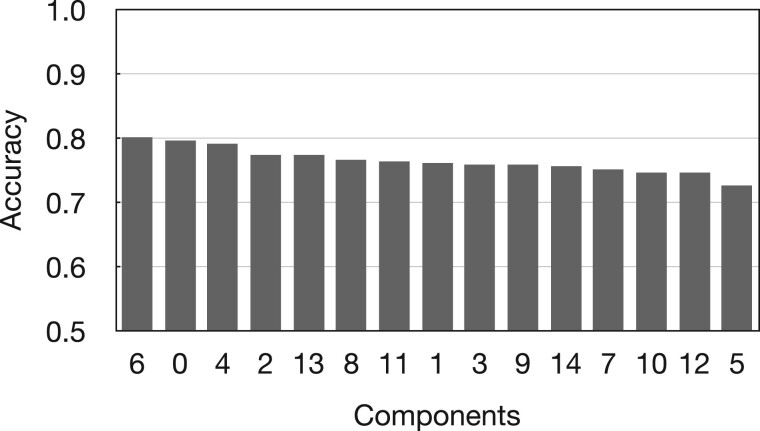
The predictive performance of each network component with NSPA-based feature transformation method. As shown in the figure, network components 0, 2, 4 and 6 are the top four epistasis network components with the best single-component predictive performance in the context of feature transformation based on NSPA

Each selected network component may have several genetic variables that can explain disease association through feature interactions. So, each network component of the comprehensive network can be considered an independent collection of genetic interactions that collectively explain disease association. The results, shown in Table 2 and Figure 4, suggest that the cross-validated predictive performances for different network components are different. Some individual network components cannot achieve equivalent predictive performance when all 15 components are considered together. LC6, LC0, LC4 and LC2 have the highest testing accuracy. The predictive performance of LC5, LC7, LC10 and LC12 is lower than the overall predictive performance. We consider the decrease in predictive performance results from the loss of disease-relevant features contained in the rest 14 network components.

**Table 2.** The predictive performance of NSPA-based feature transformation for the largest network components

LC	Precision (Case)			Recall (Case)			F1-score (Case)			Accuracy		
	Delta	Ori_LR	Ori_RF	Delta	Ori_LR	Ori_RF	Delta	Ori_LR	Ori_RF	Delta	Ori_LR	Ori_RF
0	0.829	0.581	0.561	0.807	0.524	0.549	0.817	0.550	0.553	0.795	0.517	0.504
1	0.798	0.536	0.557	0.773	0.503	0.564	0.785	0.519	0.559	0.761	0.471	0.501
2	0.816	0.568	0.580	0.780	0.537	0.565	0.796	0.551	0.572	0.774	0.505	0.520
3	0.797	0.576	0.572	0.773	0.538	0.551	0.783	0.556	0.561	0.759	0.514	0.511
4	0.820	0.579	0.592	0.812	0.534	0.588	0.815	0.554	0.589	0.791	0.515	0.536
5	0.772	0.573	0.560	0.732	0.519	0.537	0.751	0.545	0.548	0.725	0.508	0.497
6	0.838	0.589	0.615	0.804	0.558	0.589	0.820	0.572	0.602	0.800	0.529	0.557
7	0.794	0.572	0.579	0.757	0.527	0.559	0.775	0.547	0.568	0.750	0.508	0.520
8	0.799	0.556	0.594	0.786	0.522	0.599	0.791	0.538	0.596	0.765	0.493	0.542
9	0.803	0.533	0.556	0.764	0.491	0.527	0.782	0.511	0.541	0.758	0.468	0.493
10	0.789	0.586	0.612	0.756	0.510	0.570	0.772	0.545	0.589	0.747	0.517	0.552
11	0.799	0.574	0.599	0.778	0.529	0.591	0.788	0.550	0.593	0.763	0.510	0.543
12	0.791	0.565	0.591	0.757	0.497	0.599	0.773	0.529	0.594	0.747	0.498	0.538
13	0.817	0.548	0.619	0.775	0.490	0.583	0.795	0.516	0.599	0.773	0.483	0.560
14	0.799	0.562	0.598	0.760	0.487	0.561	0.779	0.521	0.578	0.755	0.495	0.538

*Notes*: *Delta* represents building predictive model using logistic regression algorithm on the dataset with feature transformation. *Ori_LR* and *Ori_RF* represent building predictive models using logistic regression and RF algorithms on the dataset without feature transformation.

Since linear regression methods cannot identify feature interactions, we also construct prediction models for the original features using the RF algorithm. The results indicate logistic regression algorithm based on the dataset with a feature transformation process outperforms the RF algorithm based on the dataset without feature transformation (Table 2).

### 3.4 Feature importance analysis

Feature transformation can improve the performance of the predictive algorithm. A subsequent analysis could identify the importance of these transformed 1035 genetic variables to the predictive outcome. The feature importance determination process based on re-sampling is defined in Section 2.6.

**Fig. 5.**
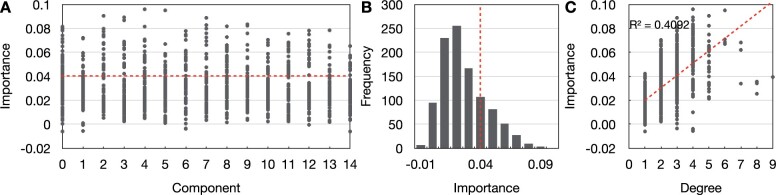
Results for feature importance analysis. (**A**) Feature importance of genetic variables grouped by components. Genetic variables that have an importance value above the red line are selected for functional enrichment analysis. (**B**) The distribution of genetic variable importance value. Genetic variables that have an importance value to the right of the red line are selected for functional enrichment analysis. (**C**) The correlation between feature importance and node degree in comprehensive network

The result suggests that all 15 network components contain important genetic variables. The most important genetic variables scatter across all network components (Fig. 5A) and have a higher degree in the comprehensive network than the less important variables (Fig. 5C). In addition, the most important genetic variables do not have significant main effects (Supplementary Table S5), indicating feature transformation method based on NSPA can identify disease-relevant genetic variables with minor individual variable effects. We submitted the rsIDs of 280 genetic variables with an importance value greater than 0.04 to g:Profiler. The g:Profiler highlights 38 terms with an adjusted *P*-value lower than 5% (Supplementary Table S3).

### 3.5 Overfitting analysis

Overfitting analysis is an important technique for evaluating predictive models. In this research, overfitting analysis is conducted to confirm the actual benefits of the feature transformation process based on NSPA.

The dataset is divided into two parts, with the training set (80%) used for constructing the comprehensive network of NSPA and feature transformation. The validation set (20%) is used for overfitting analysis. We first build prediction models for the dataset without feature transformation and consider the predictive performance as the baseline. The prediction model is established through the training folds of the 5-folds CV. The testing folds and the validation set are used to evaluate the testing and validation performance. Once this has been calculated, we move onto feature transformation based on NSPA. In this regard, we first utilize the entire training dataset to construct the comprehensive network of NSPA. The training folds of the 5-folds CV are used to perform feature transformation for all observations in the dataset. The transformed dataset will be used to build predictive models. The predictive performance of the models constructed from different paths is compared to determine the degree of overfitting of the proposed feature transformation process.

**Fig. 6.**
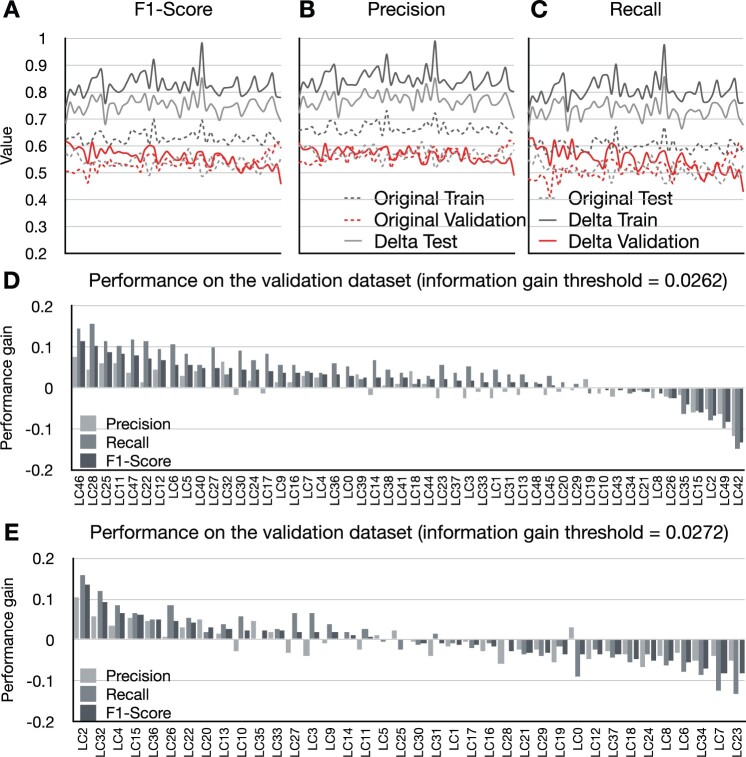
Results of the overfitting analysis and the study on the effect of information gain threshold. **A**, **B** and **C** demonstrate the impact of the feature transformation process on the performance (*y*-axis) of the predictive algorithm (logistic regression) for different network components (*x*-axis). Where the performances of the algorithm with/without feature transformation are represented using solid/dashed lines. The performance gain in **D** and **E** quantifies the improvement of predictive performance on the validation data after using the feature transformation process

We select network components (*N* = 50) containing more than 30 variables from the comprehensive network with an information gain threshold of 0.0262 for subsequent analysis. The comprehensive network with a threshold of 0.0262 contains the largest number of network components. The results suggest that although NSPA-based feature transformation improves the testing performance of the network component-specific predictive models, it suffers from significant predictive power loss over the validation dataset (see Fig. 6A). Nevertheless, the recall of diseased individuals still remained better than that of the model constructed with the original features (see Fig. 6C). As such, NSPA-based feature transformation is still a valuable step for enabling predictive models to consider feature interactions.

### 3.6 The selection of edge weight cut-off

We also conduct experiments to understand the effect of information gain threshold selection on the quality of the feature transformation process. Based on the experiment configuration of the overfitting analysis, we compare the predictive capabilities of network components from the comprehensive network with different threshold values.

Our experiments investigate two information gain thresholds. For the network configuration used for overfitting analysis, we observed that the feature transformation process can improve the predictive performance for the majority of the selected network components on the validation dataset (see Fig. 6D). However, when the threshold is set to 0.0272 (38 network components containing more than 13 variables are selected), the performance gain achieved by the feature transformation process is reduced (see Fig. 6E). We believe this is due to the decrease in the number of edges contained in the LC and the overall network. Although the edges in the network contain stronger feature interactions, they are not sufficient to compensate for the loss in the number of feature interactions, and thus the effectiveness of NSPA is diminished.

These results indicate that tuning information gain threshold can significantly influence feature transformation through changes in network topology and demonstrate how important it is for researchers to carefully adjust the parameter for successful results.

## 4 Discussion

Existing genetic-association studies concentrate on the risk impact of individual genetic variables. However, due to genetic epistasis, most disease-related genetic variables have no significant main effects. To characterize epistasis, genetic interactions have been represented as a network. With the network alone, we cannot determine the risk impact of these epistatic interactions at subject resolution. This gap leads to GWAS unable to fully leverage the knowledge provided by the network. The NSPA-based feature transformation method transforms the value of each genetic variable by considering the subject-specific risk impact of its interactions. The computational framework, illustrated in Figure 1, paves the way for subsequent predictive algorithm construction and genetic architecture discovery in the context of genetic interactions.

In this study, feature selection is performed using network investigation. We extract the largest 15 network components from the epistasis network of NSPA. These network components bring together as many as 1035 genetic variables and 1064 epistatic interactions. These network components imply the most significant biological correlations, as they are the largest components in the epistasis network. The functional enrichment analysis using g:Profiler (Raudvere *et al.*, 2019) identifies 107 important GO terms with adjusted *P*-values lower than 5%. These terms correlate to several cancer-related biological terms, such as cell development (GO:0048468) (Giannakakis *et al.*, 2008), cell adhesion (GO:0007155) (Ertel *et al.*, 2006), regulation of ion transport (GO:0043269) (Huang *et al.*, 2017) and Ras GTPase binds (GO:0030695) (Liu *et al.*, 2018). Through feature importance analysis, we identify disease-related genetic variables from the dataset processed by the feature transformation method. The rsIDs of the most important 280 genetic variables are submitted to g:Profiler. Plasma membrane (GO:0005886) is the second most significant term, which is previously correlated to the risk for CRC (Liang *et al.*, 2016).

The proposed feature transformation method helps the logistic regression algorithm to achieve 75.2% 5-fold CV testing accuracy, which outperforms the logistic regression (50.4%) and RF algorithms (51.4%) constructed directly on genetic variables without the feature transformation process. Given the most important genetic variables scattered across all network components (Fig. 5A), we further investigate the predictive performance of each network component (Fig. 4). The top four best performing network components are components LC6, LC0, LC4 and LC2 (Fig. 4). The functional enrichment analysis for the variables in each network component is reported in Supplementary Table S3.

By setting up a separate validation dataset from the NSPA process, we find that reducing the information gain threshold can help the feature transformation process to overcome overfitting. The results indicate that an excessively high information gain threshold prevents most LCs from benefiting from the feature transformation process (Fig. 6E). When more feature interactions are incorporated into the feature transformation process, the predictive performance of the machine learning method is improved (Fig. 6D). This result suggests that the number of feature interactions is more important than the strength of the interactions.

In summary, NSPA and the accompanying feature transformation method characterize the collective risk impact of genetic interactions at single-subject resolution. This framework enables the risk impact of pairwise feature interactions to be reduced to individual variables, allowing machine learning algorithms that cannot handle feature interactions to recognize the collective risk impact of feature interactions. Our results suggest that a logistic regression algorithm based on feature transformation can outperform a machine learning algorithm that considers feature interaction. Thus, the genetic architecture derived from the feature importance analysis is more persuasive than the architecture derived from the conventional approach. The success of NSPA confirms the role of genetic interaction in developing complex diseases and provides a novel approach for GWAS to identify genetic architecture in the context of epistasis.

## Supplementary Material

vbad010_Supplementary_Data

## Data Availability

The simulation datasets can be accessed through the Penn Machine Learning Benchmarks (Romano *et al.*, 2022), at https://github.com/EpistasisLab/pmlb. The GWAS data can be accessed through the CRC Transdisciplinary (CORECT) consortium (Schumacher *et al.*, 2015). The GWAS data underlying this article cannot be shared publicly due to privacy reasons.
