# Supplementary material for: NSPA: characterizing the disease association of multiple genetic interactions at single-subject resolution
Source: Bioinform Adv. 2023 Feb 7;3(1):vbad010. doi: 10.1093/bioadv/vbad010 (PMC9927570; doi:10.1093/bioadv/vbad010)
Supplement: vbad010_Supplementary_Data [file vbad010_supplementary_data.zip › vbad010_Supplementary_Data/Supplementary Materials for NSPA-characterizing the disease association of multiple genetic interactions at single-subject resolution.pdf]

Supplementary materials for

NSPA: characterizing the disease risk of multiple genetic interactions at single-subject resolution

Zhendong Sha *et al.*

Corresponding author: Ting Hu, ting.hu@queensu.ca

This PDF file includes:

- Table-S1: Results for synthetic dataset (see Table-S1.xlsx)
- Table-S2: The g:Profiler query parameters
- Table-S3: Functional enrichment analysis (see Table-S3.xlsx)
- Figure-S4: The diagram for model training procedure based on feature transformation
- Table-S5: Feature importance analysis of logistic regression model based on NSPA

Table-S2: The g:Profiler query parameters

Parameters	Value
**version**	e101_eg48_p14_baf17f0
**organism**	hsapiens
**ordered**	FALSE
**sources**	GO:MF, GO:CC, GO:BP, KEGG, REAC, HPA, CORUM, WP
**multiquery**	FALSE
**numeric ns**	ENTREZGENE_ACC
**domain scope**	annotated
**measure underrepresentation**	FALSE
**significance threshold method**	FALSE
**user threshold**	0.05
**no evidence**	FALSE
**filter results**	FALSE

Figure-S4: The diagram for model training procedure based on feature transformation

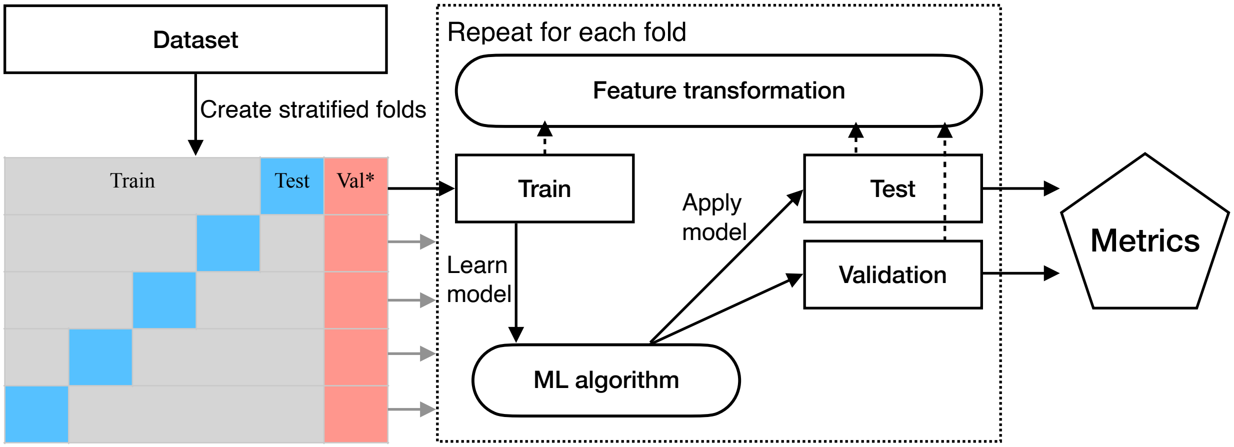

The validation fold (20%) is optional.

Table-S4 Feature importance analysis of logistic regression model based on NSPA

SNP	|Importance|	Std	Degree	Rank %	Gene
**rs6669972**	0.096	0.014	4	68.26%	
**rs4860406**	0.095	0.015	6	20.62%	ADGRL3
**rs2824722**	0.090	0.013	5	3.74%	TMPRSS15
**rs1339521**	0.089	0.013	4	38.29%	
**rs10423969**	0.089	0.014	3	28.36%	WDR88
**rs16883882**	0.087	0.014	4	18.68%	
**rs12964779**	0.083	0.012	4	9.36%	RBFA,ENSG00000267127
**rs17408765**	0.083	0.013	4	4.78%	NPAS3
**rs17235308**	0.082	0.012	5	75.70%	SLC23A4P
**rs10144013**	0.082	0.015	3	31.24%	EML1
**rs2830249**	0.081	0.012	4	27.01%	CYYR1,CYYR1-AS1
**rs2121001**	0.081	0.010	3	65.07%	
**rs3198502**	0.080	0.010	3	16.65%	STAT5A
**rs4969170**	0.080	0.016	4	21.20%	SOCS3-DT
**rs2811802**	0.080	0.012	4	56.06%	ADAMTSL1
**rs2541230**	0.078	0.012	4	15.07%	ADAMTS14
**rs713383**	0.078	0.013	4	19.41%	TRERF1
**rs4598778**	0.078	0.013	4	74.53%	LINC00547
**rs10020457**	0.078	0.011	3	11.79%	
**rs1627835**	0.077	0.012	4	46.46%	
**rs10768950**	0.077	0.013	4	49.33%	OR51B5,HBG2,HBE1,ENSG00000239920
**rs17641254**	0.076	0.013	4	22.23%	ACSF2
**rs2661665**	0.076	0.012	4	52.87%	APBB2
**rs41420347**	0.076	0.011	4	50.91%	SORCS2

SNP	|Importance|	Std	Degree	Rank %	Gene
**rs1542007**	0.075	0.009	3	57.45%	WDR7
**rs11247870**	0.075	0.014	5	55.04%	CATSPER4
**rs2836399**	0.075	0.012	3	56.57%	ERG
**rs6510057**	0.074	0.013	4	49.58%	ZNF543
**rs622219**	0.074	0.014	4	9.99%	
**rs2160643**	0.073	0.012	3	51.72%	PID1
**rs1891673**	0.073	0.011	3	22.24%	DCLK1
**rs1000040**	0.072	0.012	3	8.29%	LINC02250
**rs993871**	0.072	0.012	4	69.50%	
**rs2841292**	0.072	0.012	4	76.86%	
**rs7863282**	0.071	0.011	3	2.23%	
**rs1517700**	0.071	0.018	6	95.22%	
**rs7654754**	0.070	0.014	4	65.40%	SHROOM3
**rs16975871**	0.070	0.010	2	71.42%	
**rs322129**	0.070	0.011	3	37.61%	
**rs17456670**	0.070	0.012	3	88.55%	LINC02458
**rs7832145**	0.069	0.011	5	30.71%	JPH1
**rs2777738**	0.069	0.012	2	81.04%	
**rs12799172**	0.069	0.014	4	25.27%	GVINP1
**rs11716015**	0.068	0.015	6	65.97%	MECOM
**rs219180**	0.068	0.012	4	88.01%	LTBP1
**rs10815183**	0.068	0.014	7	2.42%	KANK1
**rs11989824**	0.068	0.014	4	78.96%	ENSG00000254277
**rs7654896**	0.067	0.011	3	68.32%	ENSG00000248373
**rs4850358**	0.067	0.011	3	62.60%	
**rs7032451**	0.067	0.013	3	81.80%	SUSD1

SNP	|Importance|	Std	Degree	Rank %	Gene
**rs12300073**	0.067	0.013	6	44.64%	ANKS1B
**rs7165303**	0.067	0.011	2	27.70%	RYR3
**rs1667550**	0.067	0.011	3	23.74%	AQP4-AS1
**rs1800764**	0.066	0.011	3	81.30%	
**rs2143734**	0.066	0.010	3	28.48%	GLP1R
**rs10934215**	0.066	0.012	3	24.24%	NEPRO-AS1,ENSG00000241219
**rs1334392**	0.066	0.012	3	1.44%	PCDH9
**rs5751416**	0.066	0.013	4	34.02%	
**rs4958645**	0.066	0.009	3	93.50%	
**rs1924208**	0.065	0.012	5	92.81%	
**rs6025642**	0.065	0.011	2	37.37%	
**rs432284**	0.065	0.011	3	11.62%	
**rs2463838**	0.065	0.011	4	1.40%	
**rs2643328**	0.065	0.014	3	58.66%	PCSK5
**rs4894196**	0.065	0.012	4	11.09%	LINC01117,ENSG00000230552
**rs234146**	0.065	0.011	4	60.06%	ENSG00000287501
**rs862334**	0.065	0.012	5	37.96%	NRXN3
**rs7779296**	0.064	0.011	3	48.36%	PMS2CL
**rs17552089**	0.064	0.014	3	45.40%	OLFM4
**rs11635194**	0.064	0.013	5	76.24%	ENSG00000259639
**rs12439952**	0.063	0.010	3	82.03%	
**rs932443**	0.063	0.013	3	84.67%	GLP1R
**rs4931635**	0.063	0.012	4	99.10%	FGD4
**rs3097563**	0.063	0.010	2	12.71%	
**rs7296335**	0.063	0.012	3	54.00%	PTPRQ

SNP	|Importance|	Std	Degree	Rank %	Gene
**rs10863457**	0.062	0.014	4	99.76%	
**rs2174076**	0.062	0.020	7	17.41%	
**rs2349415**	0.062	0.012	3	70.14%	FSHR,ENSG00000282890
**rs3913300**	0.062	0.014	5	61.40%	CNTN6
**rs13113735**	0.062	0.010	3	28.36%	
**rs3133732**	0.062	0.012	5	90.93%	CFAP418-AS1
**rs3093170**	0.062	0.014	3	40.83%	CYP4F2
**rs7024043**	0.061	0.012	3	86.67%	LINC01505
**rs11032968**	0.061	0.012	2	44.27%	OR52I2
**rs6585256**	0.061	0.011	3	59.19%	
**rs1005696**	0.061	0.012	5	48.83%	CBR1,SETD4,CBR1-AS1
**rs749613**	0.061	0.013	4	88.48%	CNTN4
**rs2215849**	0.061	0.012	4	32.77%	LINC01122
**rs10766639**	0.061	0.014	3	64.70%	
**rs182784**	0.060	0.010	3	42.60%	BMP7
**rs220278**	0.060	0.008	2	77.89%	UMODL1
**rs2059271**	0.060	0.009	2	20.34%	RBFOX1
**rs170961**	0.060	0.012	4	26.03%	
**rs4772641**	0.059	0.011	3	95.14%	
**rs10763963**	0.059	0.012	3	30.12%	
**rs2160091**	0.059	0.012	3	70.17%	LINC01122
**rs4861498**	0.059	0.011	3	24.84%	TENM3,ENSG00000248266
**rs2171209**	0.058	0.010	2	55.60%	SYTL3
**rs1220315**	0.058	0.012	3	2.21%	
**rs3814892**	0.058	0.011	3	48.91%	
**rs4757597**	0.058	0.011	3	38.72%	

SNP	|Importance|	Std	Degree	Rank %	Gene
**rs2704917**	0.058	0.009	2	4.59%	
**rs3112112**	0.058	0.011	3	98.43%	ENSG00000288941
**rs7080305**	0.058	0.013	4	14.98%	
**rs7721839**	0.058	0.015	4	37.37%	
**rs7804817**	0.057	0.009	3	94.03%	
**rs10036065**	0.057	0.011	5	61.18%	
**rs347987**	0.057	0.010	3	16.50%	
**rs12462724**	0.057	0.013	3	16.67%	SHC2
**rs1729062**	0.057	0.014	4	25.99%	NUBP1,TVP23A
**rs829997**	0.057	0.012	4	96.42%	RAB3C,ENSG00000248733
**rs4809960**	0.057	0.013	4	16.47%	CYP24A1
**rs12155595**	0.057	0.011	3	41.71%	SGCZ
**rs12508654**	0.057	0.012	3	33.14%	GABRA4
**rs518075**	0.057	0.009	2	20.96%	RNF130
**rs9595960**	0.056	0.015	3	18.22%	CYSLTR2
**rs984773**	0.056	0.011	3	46.92%	ERBB4
**rs7824718**	0.056	0.011	2	83.75%	
**rs1376842**	0.056	0.010	2	18.93%	
**rs11162634**	0.056	0.013	3	26.00%	ADGRL4
**rs12658529**	0.056	0.012	3	5.16%	LINC02196
**rs1002316**	0.056	0.013	3	52.57%	PARVA
**rs10963675**	0.056	0.012	3	85.19%	ADAMTSL1
**rs16951597**	0.056	0.011	3	60.49%	
**rs11722498**	0.056	0.012	4	80.58%	
**rs7311627**	0.055	0.014	4	23.51%	ENSG00000258254
**rs12533473**	0.055	0.011	3	71.16%	

SNP	|Importance|	Std	Degree	Rank %	Gene
**rs605008**	0.055	0.009	2	10.82%	
**rs4820478**	0.055	0.010	2	57.09%	
**rs4770433**	0.055	0.010	3	69.84%	SACS
**rs6559629**	0.055	0.011	3	80.66%	TLE1
**rs11762253**	0.055	0.010	2	3.89%	ENSG00000230333
**rs4894724**	0.055	0.011	2	52.61%	NAALADL2
**rs2709591**	0.055	0.010	2	89.73%	ASAP2
**rs150366**	0.055	0.009	2	10.95%	NLRC5
**rs10073312**	0.055	0.010	2	10.67%	
**rs3808357**	0.055	0.009	2	90.54%	ODF1
**rs11150909**	0.055	0.009	2	99.79%	
**rs12638646**	0.055	0.011	2	92.39%	ALCAM
**rs4953616**	0.054	0.012	3	41.65%	STON1-GTF2A1L,LHCGR,GTF2A1L,ENSG00000279956
**rs602117**	0.054	0.013	4	33.78%	CHRM3
**rs16934661**	0.054	0.011	3	39.88%	LRRC4C
**rs11226679**	0.054	0.011	3	97.55%	
**rs4750058**	0.054	0.012	4	84.74%	
**rs7431217**	0.054	0.012	4	52.16%	
**rs1003710**	0.054	0.011	4	25.00%	SMAP2
**rs10448137**	0.054	0.011	2	94.94%	SH2D4A
**rs7675387**	0.054	0.012	4	33.47%	NPFFR2
**rs4979467**	0.054	0.012	3	3.63%	DELEC1
**rs685163**	0.054	0.012	3	20.76%	SEZ6L
**rs2961851**	0.054	0.008	2	9.17%	
**rs7949874**	0.053	0.013	3	35.51%	

SNP	|Importance|	Std	Degree	Rank %	Gene
**rs469280**	0.053	0.011	4	8.49%	
**rs11871217**	0.053	0.011	2	26.18%	NMT1,ENSG00000289024
**rs17558560**	0.053	0.013	3	31.02%	KRT27
**rs16940062**	0.053	0.010	2	40.07%	ST8SIA5-DT
**rs2307019**	0.053	0.009	3	45.45%	IZUMO1
**rs6047163**	0.052	0.015	5	61.21%	STK35
**rs17803986**	0.052	0.010	3	20.26%	
**rs10786828**	0.052	0.009	3	22.92%	SORCS3
**rs10865864**	0.052	0.016	4	32.99%	ENSG00000223727
**rs12516704**	0.052	0.012	3	85.27%	
**rs4555392**	0.052	0.010	2	52.08%	
**rs3801839**	0.052	0.012	3	4.92%	SKAP2
**rs2431124**	0.051	0.009	2	21.88%	TBC1D9B
**rs708271**	0.051	0.011	5	84.85%	NLRC5
**rs6447976**	0.051	0.009	2	9.07%	PACRGL,KCNIP4
**rs6785310**	0.051	0.011	3	50.13%	XXYLT1
**rs7786437**	0.051	0.010	2	74.26%	
**rs7592100**	0.051	0.009	2	18.67%	
**rs1873250**	0.051	0.011	4	30.04%	GRM4
**rs529386**	0.050	0.010	2	88.16%	
**rs84191**	0.050	0.013	3	86.98%	
**rs12987292**	0.050	0.012	3	9.90%	PARD3B
**rs745321**	0.050	0.011	2	86.56%	
**rs4309706**	0.050	0.011	2	23.87%	LINC02069
**rs13150331**	0.050	0.011	3	98.43%	ENSG00000250771
**rs10002760**	0.050	0.010	2	17.56%	

SNP	|Importance|	Std	Degree	Rank %	Gene
**rs1465677**	0.049	0.010	2	15.87%	KLHL29
**rs13176940**	0.049	0.010	3	25.46%	PDE4D
**rs5916050**	0.049	0.010	3	41.53%	
**rs1590359**	0.049	0.011	2	53.15%	TSTD3
**rs404619**	0.049	0.009	2	46.47%	CDH13,HSBP1, ENSG00000260788
**rs2817431**	0.049	0.010	4	90.07%	
**rs10004243**	0.049	0.010	3	2.44%	TTC29
**rs6006611**	0.049	0.011	3	48.75%	SAMM50,PARVB
**rs912438**	0.049	0.010	2	53.77%	
**rs919178**	0.049	0.015	4	6.90%	SP110
**rs10062069**	0.048	0.011	2	90.64%	FER
**rs753017**	0.048	0.010	2	91.70%	EZR
**rs12959006**	0.048	0.010	2	99.55%	MBP
**rs967582**	0.048	0.010	2	90.51%	ELAVL4
**rs4601989**	0.048	0.012	4	19.14%	SMAD3
**rs11125039**	0.048	0.009	2	80.05%	PRKCE
**rs6569099**	0.048	0.009	2	69.24%	
**rs567578**	0.048	0.010	2	15.48%	ENSG00000285517
**rs1544846**	0.048	0.012	2	28.99%	PXDN
**rs7084875**	0.048	0.009	2	66.95%	TCF7L2
**rs12450446**	0.048	0.013	5	82.86%	ASIC2
**rs17756404**	0.048	0.014	4	7.11%	RAD51B
**rs7097552**	0.048	0.012	3	54.21%	
**rs32494**	0.048	0.010	2	77.48%	
**rs7014579**	0.048	0.009	2	35.58%	TDRP
**rs4736796**	0.048	0.014	3	32.51%	

SNP	|Importance|	Std	Degree	Rank %	Gene
**rs10924730**	0.048	0.010	3	78.62%	SMYD3
**rs12752888**	0.048	0.010	3	98.09%	
**rs4129294**	0.048	0.014	3	43.07%	
**rs3807848**	0.048	0.010	2	56.30%	ICA1
**rs11781147**	0.048	0.012	4	41.89%	FER1L6
**rs10836964**	0.047	0.010	3	88.05%	
**rs10875652**	0.047	0.012	4	67.68%	
**rs13201016**	0.047	0.009	3	98.99%	
**rs1750961**	0.047	0.011	2	13.32%	PKHD1
**rs7365286**	0.047	0.009	2	43.91%	
**rs2047087**	0.047	0.010	2	15.58%	ENSG00000259692
**rs6915319**	0.047	0.013	3	70.99%	ZDHHC14
**rs10214212**	0.046	0.010	2	52.57%	TENM2,ENSG00000253925
**rs2134477**	0.046	0.011	2	93.48%	SKINT1L
**rs1348029**	0.046	0.010	2	20.40%	
**rs1078739**	0.046	0.014	5	58.51%	
**rs17011024**	0.046	0.010	3	42.45%	ARHGAP24
**rs17538843**	0.046	0.011	3	81.60%	
**rs11033160**	0.046	0.013	3	50.72%	
**rs7332030**	0.046	0.010	2	79.08%	
**rs576598**	0.046	0.011	2	14.20%	DDX25,ENSG00000255027
**rs10489636**	0.046	0.011	3	45.41%	CD48,ENSG00000228863
**rs6609094**	0.045	0.008	2	96.25%	
**rs761167**	0.045	0.011	3	60.81%	SLC25A20P1
**rs7118075**	0.045	0.013	2	57.57%	SLC17A6-DT
**rs7145143**	0.045	0.012	3	75.78%	

SNP	|Importance|	Std	Degree	Rank %	Gene
**rs12315573**	0.045	0.011	4	10.28%	LINC02384
**rs7147607**	0.045	0.012	4	59.19%	UNC79
**rs7538619**	0.045	0.014	4	17.04%	
**rs7141420**	0.045	0.009	2	3.34%	NRXN3
**rs1116986**	0.045	0.015	4	27.54%	C8orf34
**rs1806722**	0.045	0.014	4	21.07%	
**rs7629818**	0.045	0.011	3	13.47%	FRMD4B
**rs11934369**	0.045	0.009	2	23.46%	ENSG00000279460
**rs17355157**	0.045	0.010	3	78.71%	ENSG00000254687
**rs2898691**	0.044	0.012	3	21.42%	NIFKP6,LIM2-AS1
**rs6545750**	0.044	0.011	2	65.05%	ENSG00000271955
**rs10503098**	0.044	0.012	4	51.76%	
**rs3092043**	0.044	0.010	2	63.82%	PTPRT
**rs10093390**	0.044	0.009	2	15.46%	
**rs7172127**	0.044	0.013	3	2.74%	PCSK6
**rs1167432**	0.044	0.010	2	54.52%	
**rs1550720**	0.044	0.012	5	0.88%	PRKG1
**rs1517150**	0.044	0.011	2	9.09%	RBMS3,RBMS3-AS3,ENSG00000283563
**rs12622722**	0.044	0.010	2	94.33%	COL6A3
**rs1033411**	0.044	0.011	2	23.92%	
**rs1152399**	0.043	0.011	3	13.29%	
**rs1231056**	0.043	0.010	2	64.40%	
**rs2675181**	0.043	0.009	2	53.57%	SRGAP3
**rs7938684**	0.043	0.012	2	19.65%	TENM4,ENSG00000255345

SNP	|Importance|	Std	Degree	Rank %	Gene
**rs328794**	0.043	0.009	2	18.22%	
**rs7085352**	0.042	0.012	3	72.12%	LRMDA
**rs2554682**	0.042	0.010	2	87.38%	CSMD1
**rs8106664**	0.042	0.010	2	40.78%	SLC44A2
**rs3807852**	0.042	0.010	2	18.58%	ICA1
**rs5906992**	0.042	0.011	1	41.82%	
**rs11092578**	0.042	0.014	4	2.88%	MORC4
**rs4329483**	0.042	0.010	2	78.09%	PDE4B
**rs869894**	0.042	0.010	2	28.35%	
**rs796140**	0.042	0.009	2	71.72%	
**rs6913163**	0.041	0.010	2	3.56%	
**rs7876414**	0.041	0.011	3	99.89%	
**rs17052811**	0.041	0.012	3	35.96%	TPD52L1
**rs7315885**	0.041	0.010	3	40.57%	TMEM132D
**rs12522315**	0.041	0.013	3	61.17%	
**rs1064103**	0.041	0.012	3	25.45%	ADGRE2
**rs1539438**	0.041	0.013	2	88.23%	AP4B1-AS1
**rs16852535**	0.041	0.009	2	51.27%	ENSG00000244137
**rs6822207**	0.041	0.011	2	96.83%	MAML3
**rs38247**	0.041	0.009	2	36.61%	
**rs4067228**	0.041	0.013	3	74.64%	
**rs4782872**	0.040	0.010	2	97.52%	
**rs1546746**	0.040	0.009	2	28.37%	TRHDE
**rs6865709**	0.040	0.012	3	85.86%	LINC02240
**rs12146686**	0.040	0.011	2	71.44%	OR52I2
**rs17739271**	0.040	0.007	1	66.06%	DYNLRB2-AS1
**rs1165956**	0.040	0.011	5	15.32%	

SNP	|Importance|	Std	Degree	Rank %	Gene
**rs493742**	0.040	0.009	2	39.77%	ENSG00000233290
**rs6729809**	0.040	0.009	2	53.76%	STON1-GTF2A1L,LHCGR,GTF2A1L,ENSG00000279956
**rs4233728**	0.040	0.010	2	97.70%	PPP1CB
**rs4936742**	0.040	0.012	3	15.09%	UBASH3B,ENSG00000285909
**rs6874307**	0.040	0.010	3	28.76%	
**rs12893940**	0.040	0.012	3	55.06%	
**rs12264785**	0.039	0.012	3	40.28%	ENSG00000287277
**rs17105411**	0.039	0.009	9	86.57%	TMEM63C,ENSG00000259164
**rs7949993**	0.039	0.011	2	0.69%	
**rs4736695**	0.039	0.012	3	91.47%	ST3GAL1
**rs2193414**	0.039	0.010	2	73.99%	NRXN1
**rs11126163**	0.039	0.010	2	47.39%	
**rs10159966**	0.039	0.010	2	1.78%	
**rs10984029**	0.039	0.016	3	25.56%	
**rs7790976**	0.039	0.010	2	15.24%	TMEM178B
**rs872113**	0.039	0.008	2	49.24%	
**rs7127438**	0.039	0.009	2	49.36%	JRKL,JRKL-AS1
**rs10043614**	0.039	0.011	2	13.94%	LINC01947
**rs2992736**	0.039	0.010	3	89.04%	
**rs8106205**	0.039	0.009	3	47.95%	
**rs4945097**	0.039	0.010	2	30.43%	ENSG00000236304
**rs17045852**	0.039	0.008	1	57.43%	
**rs1341473**	0.039	0.009	2	41.82%	ENSG00000286746

SNP	|Importance|	Std	Degree	Rank %	Gene
**rs1702468**	0.039	0.011	3	76.82%	
**rs6567313**	0.039	0.011	2	4.99%	
**rs2249380**	0.038	0.011	2	80.76%	DCDC2C
**rs9492432**	0.038	0.011	2	16.19%	
**rs7548057**	0.038	0.009	2	60.61%	
**rs16868536**	0.038	0.011	2	85.19%	TTC23L
**rs7294907**	0.038	0.010	2	64.76%	
**rs3846075**	0.038	0.011	2	60.23%	LINC02006
**rs6507838**	0.038	0.011	2	61.60%	ZBTB7C
**rs10891845**	0.038	0.009	1	36.11%	
**rs710681**	0.038	0.010	2	30.39%	PRANCR
**rs17259985**	0.038	0.011	3	70.01%	FAM107B
**rs8082149**	0.038	0.011	2	71.44%	ANKFN1
**rs10200706**	0.038	0.010	2	71.18%	PID1
**rs738809**	0.038	0.009	2	33.39%	
**rs6619657**	0.038	0.010	3	96.10%	
**rs418874**	0.038	0.010	2	51.13%	
**rs7853782**	0.038	0.011	2	33.35%	OLFM1
**rs952973**	0.037	0.010	2	57.81%	
**rs7586507**	0.037	0.013	4	71.86%	LINC01954
**rs3783295**	0.037	0.009	2	71.47%	LINC01551
**rs1783919**	0.037	0.011	3	39.80%	NFRKB
**rs1950192**	0.037	0.011	3	64.72%	LINC01551
**rs7105484**	0.037	0.012	2	33.29%	
**rs1263811**	0.037	0.010	2	58.74%	SALL2
**rs11937236**	0.037	0.013	4	29.47%	TENM3
**rs1256719**	0.037	0.011	2	39.23%	

SNP	|Importance|	Std	Degree	Rank %	Gene
**rs2830201**	0.037	0.011	3	31.08%	CYYR1-AS1,ENSG00000232692
**rs4402086**	0.037	0.014	4	19.04%	PLA2G4A
**rs3002130**	0.036	0.011	3	18.27%	
**rs11994674**	0.036	0.009	2	86.02%	
**rs1492087**	0.036	0.010	2	7.16%	CPB1
**rs7650856**	0.036	0.012	2	47.75%	KCNMB2,ENSG00000223930
**rs10775299**	0.036	0.014	4	23.09%	
**rs10834318**	0.036	0.012	3	40.19%	
**rs13180395**	0.036	0.010	2	8.34%	
**rs9545836**	0.036	0.011	2	20.39%	
**rs13255574**	0.036	0.013	4	71.93%	ANGPT2,MCPH1
**rs7405957**	0.036	0.014	2	56.83%	B3GNTL1
**rs7630830**	0.036	0.009	2	35.66%	ARHGEF3
**rs8039278**	0.036	0.011	2	52.53%	
**rs2876761**	0.036	0.013	4	27.04%	ENSG00000286746
**rs3762830**	0.036	0.008	1	16.16%	RNF4
**rs8063688**	0.036	0.011	2	34.27%	CDYL2
**rs10520276**	0.036	0.012	2	14.63%	FBXO8
**rs1372120**	0.036	0.013	8	93.21%	CERKL
**rs7147170**	0.035	0.007	1	61.87%	EGLN3
**rs26880**	0.035	0.010	2	22.70%	
**rs7289071**	0.035	0.007	1	8.61%	EPIC1
**rs17799849**	0.035	0.013	3	47.54%	
**rs4252087**	0.035	0.008	2	52.32%	PLG
**rs713061**	0.035	0.010	2	29.60%	ABCA13

SNP	|Importance|	Std	Degree	Rank %	Gene
**rs7793323**	0.035	0.010	2	76.44%	TFEC
**rs2326371**	0.035	0.009	2	76.53%	
**rs10508204**	0.035	0.009	2	96.88%	IDI2-AS1
**rs2526614**	0.035	0.013	3	38.45%	TWIST1
**rs10753234**	0.035	0.007	1	54.33%	
**rs11891876**	0.035	0.014	5	52.70%	FEZ2
**rs11642137**	0.035	0.013	3	20.27%	ENSG00000261818
**rs3781638**	0.035	0.010	2	73.33%	MTNR1B
**rs7212582**	0.035	0.009	2	85.45%	ENSG00000261848
**rs4871791**	0.034	0.009	2	29.11%	CASC8
**rs790346**	0.034	0.010	2	12.10%	DLG2
**rs987534**	0.034	0.010	2	60.92%	
**rs9897212**	0.034	0.007	1	73.44%	
**rs11744003**	0.034	0.011	2	62.64%	CYFIP2,ENSG00000248544,ENSG00000285868
**rs17742319**	0.034	0.007	1	19.95%	
**rs4741218**	0.034	0.009	2	96.07%	
**rs16888555**	0.034	0.012	8	54.39%	ENSG00000271945
**rs6763361**	0.034	0.010	2	89.82%	HRG-AS1,ENSG00000283149
**rs2919381**	0.034	0.008	2	31.45%	NRG1
**rs1558697**	0.034	0.009	2	49.29%	MAGI2
**rs6085661**	0.034	0.010	4	72.77%	
**rs11622263**	0.034	0.006	1	30.00%	MEG3
**rs1025949**	0.034	0.013	7	10.32%	
**rs13228793**	0.034	0.008	1	83.64%	DPY19L1P1

SNP	|Importance|	Std	Degree	Rank %	Gene
**rs7669556**	0.034	0.010	2	6.37%	
**rs9450448**	0.034	0.007	1	1.65%	
**rs3826293**	0.034	0.011	5	86.75%	RPL23
**rs6442020**	0.034	0.007	1	37.77%	
**rs11211590**	0.034	0.010	2	63.72%	LINC01738
**rs4711374**	0.034	0.008	2	27.58%	GRM4
**rs776840**	0.033	0.008	2	38.52%	
**rs4846483**	0.033	0.008	2	86.54%	
**rs4238009**	0.033	0.008	1	79.74%	LINC02417
**rs857230**	0.033	0.007	1	59.37%	LINC02295,ENSG00000259097
**rs6555860**	0.033	0.015	3	5.15%	
**rs10802934**	0.033	0.007	1	31.16%	RGS7
**rs9309322**	0.033	0.007	1	90.80%	
**rs7874842**	0.033	0.009	2	67.76%	BNC2
**rs10815398**	0.033	0.009	2	88.84%	
**rs2018771**	0.033	0.012	3	41.94%	
**rs4246309**	0.033	0.010	2	6.22%	ADAMTS17
**rs10503428**	0.033	0.007	2	20.14%	LINC03019,LINC00681
**rs2368785**	0.032	0.010	2	90.92%	IQSEC3
**rs3013462**	0.032	0.010	3	25.49%	SMAP2
**rs35806**	0.032	0.009	2	16.04%	KCNMA1
**rs7197319**	0.032	0.010	2	4.27%	
**rs8108774**	0.032	0.012	4	82.60%	
**rs35869320**	0.032	0.008	5	53.19%	ENSG00000236230
**rs17037864**	0.032	0.007	1	70.47%	C4orf45
**rs11874505**	0.032	0.010	2	55.83%	

SNP	|Importance|	Std	Degree	Rank %	Gene
**rs12162233**	0.032	0.008	1	7.04%	FBN3
**rs4760653**	0.032	0.009	2	42.14%	SLC48A1
**rs2407246**	0.032	0.009	2	32.22%	CYSLTR2
**rs6689839**	0.032	0.010	2	31.17%	UTP25
**rs1653098**	0.032	0.011	3	94.69%	BLOC1S5-TXNDC5
**rs1825651**	0.032	0.009	2	95.11%	
**rs947367**	0.032	0.008	2	13.79%	
**rs9595945**	0.032	0.007	2	82.14%	
**rs2896243**	0.032	0.012	2	22.64%	PRIMA1
**rs7522460**	0.032	0.010	2	63.11%	
**rs651284**	0.032	0.012	3	52.56%	CDON
**rs415229**	0.032	0.009	2	94.55%	EPB41L4A
**rs2215781**	0.032	0.009	2	38.23%	
**rs547177**	0.032	0.010	2	20.24%	TMEM163
**rs12171762**	0.032	0.012	3	93.08%	
**rs6665839**	0.032	0.008	2	78.14%	AGBL4
**rs11626641**	0.032	0.008	1	0.08%	C14orf132
**rs12104693**	0.032	0.015	3	20.78%	PECR,MREG
**rs9920181**	0.031	0.011	3	18.60%	PCSK6
**rs4979892**	0.031	0.010	2	37.25%	KCNMA1
**rs1948080**	0.031	0.009	2	28.60%	ENSG00000285784
**rs7966456**	0.031	0.008	1	10.19%	SLC2A13,C12orf40
**rs12207056**	0.031	0.012	3	63.57%	MCM3
**rs6051490**	0.031	0.008	1	34.17%	TRIB3
**rs1896880**	0.031	0.010	2	65.03%	
**rs6992898**	0.031	0.008	1	90.25%	

SNP	|Importance|	Std	Degree	Rank %	Gene
**rs6818978**	0.031	0.010	2	19.47%	LINC02357
**rs8023253**	0.031	0.009	2	53.89%	
**rs749008**	0.031	0.007	1	73.63%	BIN1
**rs4903249**	0.031	0.010	2	69.16%	
**rs9809165**	0.031	0.009	2	75.41%	
**rs10143148**	0.031	0.006	1	63.16%	ENSG00000258526
**rs6981281**	0.031	0.011	2	36.17%	MTMR7,VPS37A
**rs10860299**	0.031	0.011	2	87.21%	ENSG00000258312
**rs12928583**	0.031	0.013	4	53.41%	
**rs8043049**	0.030	0.005	1	5.90%	DNAAF4,DNAAF4-CCPG1
**rs10507901**	0.030	0.009	2	59.62%	ENSG00000284196
**rs6967915**	0.030	0.010	2	23.17%	
**rs2276378**	0.030	0.009	2	4.62%	PTPN2
**rs17146997**	0.030	0.009	2	88.94%	STEAP1B
**rs1294085**	0.030	0.010	2	21.31%	DNAAF6
**rs7524973**	0.030	0.009	2	55.26%	
**rs6777142**	0.030	0.010	2	64.04%	MECOM
**rs2974039**	0.030	0.008	1	73.14%	
**rs10841496**	0.030	0.007	1	24.59%	PDE3A,PDE3A-AS1
**rs9366702**	0.030	0.010	4	37.68%	
**rs10987613**	0.030	0.010	2	8.14%	GARNL3
**rs9298146**	0.030	0.011	3	33.83%	
**rs10939067**	0.030	0.010	2	49.04%	ENSG00000248545
**rs6853596**	0.030	0.010	2	22.21%	
**rs7142232**	0.030	0.008	1	82.33%	CDKL1

SNP	|Importance|	Std	Degree	Rank %	Gene
**rs6031944**	0.030	0.010	5	72.30%	STK4
**rs14389**	0.030	0.007	2	33.70%	TRIP13
**rs886658**	0.030	0.008	1	72.92%	
**rs893740**	0.029	0.007	1	24.85%	
**rs1889625**	0.029	0.007	1	94.61%	SAMD3
**rs10946724**	0.029	0.009	1	66.62%	LINC02828
**rs927204**	0.029	0.007	1	49.41%	ABCB10
**rs12584385**	0.029	0.013	3	9.38%	
**rs381451**	0.029	0.011	2	5.49%	
**rs4742449**	0.029	0.008	1	50.19%	
**rs4884317**	0.029	0.013	3	7.64%	
**rs7999084**	0.029	0.013	2	84.96%	
**rs10095896**	0.029	0.010	2	84.22%	
**rs3012675**	0.029	0.007	1	14.14%	RFX3
**rs6756596**	0.029	0.011	2	58.59%	
**rs7864036**	0.029	0.008	1	84.74%	
**rs11676348**	0.029	0.007	1	50.11%	
**rs5751950**	0.029	0.012	3	27.67%	
**rs7667423**	0.029	0.011	2	50.86%	
**rs11769915**	0.029	0.008	2	24.28%	
**rs3775775**	0.029	0.010	2	85.66%	SULT1E1
**rs3008204**	0.029	0.006	1	99.49%	ENSG00000228255
**rs11623043**	0.029	0.011	2	91.59%	
**rs16892406**	0.029	0.009	2	9.87%	
**rs554743**	0.029	0.009	2	65.98%	ADAM33
**rs910125**	0.028	0.008	1	97.03%	

SNP	|Importance|	Std	Degree	Rank %	Gene
**rs2103273**	0.028	0.007	1	48.99%	ENSG00000249406,ENSG00000263176
**rs16867926**	0.028	0.014	2	38.76%	
**rs1275501**	0.028	0.009	4	41.33%	
**rs12217848**	0.028	0.008	1	1.16%	LINC00595,ENSG00000282863
**rs4252125**	0.028	0.010	2	32.27%	PLG
**rs1105621**	0.028	0.006	1	69.92%	HMCN2
**rs13196874**	0.028	0.008	1	55.17%	ENSG00000260604
**rs4795629**	0.028	0.009	2	79.50%	
**rs17361055**	0.028	0.010	2	92.42%	
**rs11082726**	0.028	0.011	2	45.77%	DYM-AS1
**rs17742882**	0.028	0.009	2	26.76%	DLG2
**rs862427**	0.028	0.009	2	51.07%	DNAH8
**rs2090394**	0.028	0.010	2	87.12%	DGKG
**rs11002088**	0.028	0.010	2	16.11%	KCNMA1
**rs1001793**	0.028	0.007	1	10.36%	TNFRSF10B
**rs571497**	0.028	0.009	2	48.17%	
**rs17819108**	0.028	0.006	1	5.37%	CCPG1,DNAAF4-CCPG1
**rs8033503**	0.028	0.007	1	4.30%	TRPM1
**rs9668333**	0.028	0.011	2	65.41%	LINC00937
**rs3777969**	0.028	0.010	2	16.38%	ROS1,ENSG00000282218
**rs12172194**	0.027	0.007	1	60.64%	
**rs12239846**	0.027	0.008	1	1.08%	ELAPOR1
**rs3893315**	0.027	0.011	2	83.49%	NCMAP
**rs3845787**	0.027	0.010	2	5.88%	CDC42EP3
**rs7260668**	0.027	0.011	2	59.48%	

SNP	|Importance|	Std	Degree	Rank %	Gene
**rs7073111**	0.027	0.012	5	91.92%	
**rs2803602**	0.027	0.009	2	49.35%	
**rs36044**	0.027	0.011	3	90.23%	SH3TC2
**rs135392**	0.027	0.011	2	1.87%	SHISAL1
**rs12614525**	0.027	0.010	3	64.06%	LINC02934
**rs1884361**	0.027	0.011	2	15.62%	NRG3
**rs16879814**	0.027	0.009	2	23.06%	NRG1
**rs9505014**	0.027	0.011	2	97.49%	ENSG00000271727,ENSG00000287293
**rs3127061**	0.027	0.014	2	1.06%	SGK2,ENSG00000277611
**rs4803045**	0.027	0.007	1	27.64%	SARS2,ENSG00000269547
**rs35125077**	0.027	0.009	2	87.41%	APBB2
**rs11849344**	0.027	0.009	2	26.00%	RAD51B
**rs8101563**	0.027	0.010	2	59.76%	PPP5D1P
**rs9505309**	0.027	0.010	2	26.94%	BLOC1S5-TXNDC5
**rs357936**	0.027	0.008	2	27.18%	
**rs4072819**	0.027	0.009	1	28.64%	
**rs6978606**	0.027	0.011	2	83.37%	
**rs196604**	0.027	0.007	1	83.90%	EEPD1
**rs6988624**	0.027	0.010	2	29.59%	LINC01414
**rs12441344**	0.027	0.009	2	17.71%	SMAD3
**rs1978181**	0.027	0.011	2	61.48%	
**rs1168581**	0.027	0.007	1	44.88%	
**rs1504578**	0.027	0.008	1	75.65%	ASIC2
**rs3810965**	0.027	0.009	2	78.60%	KNDC1
**rs12591766**	0.027	0.012	3	13.97%	

SNP	|Importance|	Std	Degree	Rank %	Gene
**rs16901346**	0.027	0.009	2	22.17%	
**rs8103611**	0.026	0.008	2	87.77%	VAV1
**rs6773772**	0.026	0.008	1	51.23%	OSBPL10
**rs12830004**	0.026	0.010	2	62.44%	
**rs17701547**	0.026	0.010	2	80.16%	WASL
**rs7648926**	0.026	0.013	3	59.36%	
**rs17245271**	0.026	0.013	3	86.83%	ENSG00000285679
**rs10836309**	0.026	0.007	1	91.47%	C11orf40
**rs10150274**	0.026	0.010	2	90.92%	ENSG00000258402
**rs4952280**	0.026	0.007	1	44.89%	LTBP1
**rs4543871**	0.026	0.009	1	95.61%	ATF3
**rs12232384**	0.026	0.008	1	84.55%	ENSG00000285040
**rs10215031**	0.026	0.015	2	29.00%	SDK1
**rs10498494**	0.026	0.011	2	17.48%	
**rs10493055**	0.026	0.011	2	5.02%	CSMD2
**rs1131445**	0.026	0.010	2	46.05%	STARD5,IL16
**rs3775753**	0.026	0.013	3	50.12%	CSN3
**rs469522**	0.026	0.008	1	7.01%	FCHSD1
**rs190108**	0.026	0.010	2	90.69%	
**rs926141**	0.026	0.010	2	77.27%	FHIT
**rs2074129**	0.026	0.009	1	75.35%	DOCK4
**rs1547578**	0.026	0.010	2	65.13%	CCDC149
**rs11048998**	0.026	0.010	2	13.52%	ARNTL2,ARNTL2-AS1
**rs2008765**	0.026	0.008	2	71.81%	
**rs6037811**	0.026	0.009	2	39.42%	
**rs11009265**	0.025	0.008	1	12.97%	

SNP	|Importance|	Std	Degree	Rank %	Gene
**rs9564317**	0.025	0.007	1	16.30%	PCDH9
**rs1879265**	0.025	0.010	8	35.37%	THRA
**rs854151**	0.025	0.009	1	52.72%	HDC
**rs7061912**	0.025	0.007	1	53.32%	MAMLD1
**rs1560434**	0.025	0.010	2	47.72%	
**rs10438355**	0.025	0.009	2	6.20%	
**rs3907134**	0.025	0.011	2	60.48%	
**rs1160309**	0.025	0.009	2	13.08%	
**rs17160152**	0.025	0.012	4	57.69%	
**rs11046975**	0.025	0.011	3	99.00%	SOX5
**rs4850702**	0.025	0.011	2	83.55%	HECW2
**rs2046397**	0.025	0.011	2	39.36%	MSRA
**rs3849463**	0.025	0.011	2	25.51%	
**rs1378613**	0.025	0.007	1	61.89%	
**rs11869433**	0.025	0.013	3	75.72%	ENSG00000230647
**rs7303475**	0.025	0.010	2	16.41%	ENSG00000249753
**rs2172915**	0.025	0.008	4	68.08%	ENSG00000236230
**rs1026005**	0.025	0.007	1	12.03%	MAST4
**rs567229**	0.025	0.010	2	14.17%	PDLIM1
**rs7729723**	0.025	0.011	2	88.70%	
**rs1287271**	0.025	0.008	1	49.27%	UGT3A2
**rs905858**	0.025	0.009	1	98.30%	
**rs12196394**	0.025	0.008	1	8.11%	PDE10A
**rs13354798**	0.025	0.008	3	70.23%	HCN1
**rs869789**	0.025	0.010	2	70.73%	FXYD2,ENSG00000254844,FXYD6-FXYD2

SNP	|Importance|	Std	Degree	Rank %	Gene
**rs1009478**	0.025	0.007	1	52.46%	OSBPL8
**rs10513453**	0.025	0.007	1	49.63%	LINC02006,ENSG00000243486
**rs10156134**	0.025	0.010	2	45.97%	
**rs10744030**	0.025	0.009	1	48.43%	
**rs11687338**	0.025	0.007	1	16.20%	
**rs2237831**	0.024	0.007	1	36.85%	PDGFRL
**rs10764442**	0.024	0.008	1	63.38%	KIAA1217
**rs7127594**	0.024	0.008	1	4.90%	
**rs4370755**	0.024	0.009	4	65.82%	
**rs2679261**	0.024	0.010	2	4.05%	CARD11
**rs995805**	0.024	0.008	1	93.92%	
**rs11628843**	0.024	0.007	1	98.85%	SAMD4A,SAMD4A-AS1
**rs646358**	0.024	0.008	1	34.16%	DCPS
**rs12889238**	0.024	0.010	2	79.87%	
**rs3863238**	0.024	0.007	1	77.35%	
**rs4532119**	0.024	0.009	1	39.00%	
**rs9599848**	0.024	0.007	1	93.16%	DACH1
**rs11581894**	0.024	0.006	1	99.04%	SRSF11
**rs4853420**	0.024	0.011	2	71.62%	
**rs12152004**	0.024	0.010	1	22.09%	RUNX1
**rs6822671**	0.024	0.011	2	83.27%	PCDH7
**rs955514**	0.024	0.010	2	37.11%	PLEKHA6
**rs795302**	0.024	0.007	1	94.29%	SUMF1
**rs2061201**	0.024	0.011	2	46.31%	CLRN1-AS1,ENSG00000243273,ENSG00000260234
**rs4240328**	0.024	0.011	2	68.32%	LINC02266

SNP	|Importance|	Std	Degree	Rank %	Gene
**rs174472**	0.024	0.009	2	10.55%	RAB3IL1
**rs4913353**	0.024	0.011	2	99.99%	GRIP1
**rs4988235**	0.024	0.010	3	93.27%	MCM6
**rs892031**	0.024	0.007	1	71.00%	
**rs911707**	0.024	0.007	1	70.39%	HABP2
**rs6621944**	0.024	0.006	1	91.84%	IL1RAPL2
**rs7535790**	0.023	0.007	1	36.53%	ZFP69B
**rs3898050**	0.023	0.013	3	39.13%	ENSG00000288588
**rs781707**	0.023	0.010	2	93.09%	
**rs1861870**	0.023	0.007	1	59.53%	LINC02463
**rs2224382**	0.023	0.010	2	50.11%	EYA4
**rs2179095**	0.023	0.010	4	24.69%	
**rs968021**	0.023	0.010	2	74.52%	
**rs9871288**	0.023	0.011	2	2.77%	
**rs9309089**	0.023	0.008	1	13.87%	
**rs12040249**	0.023	0.008	2	9.33%	REN
**rs378609**	0.023	0.010	2	20.38%	
**rs6428580**	0.023	0.010	4	68.35%	
**rs11605105**	0.023	0.008	1	62.61%	
**rs11216028**	0.023	0.011	2	31.33%	
**rs12463667**	0.023	0.007	1	72.44%	
**rs4790**	0.023	0.007	1	94.23%	PCBP2
**rs1893152**	0.023	0.007	1	11.54%	
**rs710377**	0.023	0.005	2	60.95%	
**rs4402864**	0.023	0.014	5	23.54%	
**rs6765905**	0.023	0.007	1	91.30%	
**rs12406019**	0.023	0.013	2	75.08%	

SNP	|Importance|	Std	Degree	Rank %	Gene
**rs17364546**	0.023	0.011	2	29.27%	LINC02566,ENSG00000256321,LINC02955
**rs8033582**	0.023	0.010	2	89.50%	ENSG00000258676
**rs7235205**	0.023	0.006	1	24.92%	
**rs17187772**	0.023	0.007	1	21.24%	LINC01915
**rs710384**	0.023	0.006	2	59.86%	
**rs10514261**	0.023	0.010	2	93.19%	
**rs4305424**	0.023	0.008	2	11.86%	WDR49
**rs1285740**	0.022	0.008	2	9.51%	
**rs136094**	0.022	0.006	1	39.59%	TBC1D22A
**rs7554186**	0.022	0.009	1	71.67%	ENSG00000237588,ENSG00000285570
**rs7281317**	0.022	0.011	2	67.10%	ENSG00000235609,LINC02246
**rs16177**	0.022	0.007	1	53.95%	
**rs6662998**	0.022	0.007	1	18.31%	LINC01755,ENSG00000234810
**rs13138544**	0.022	0.007	1	17.37%	ARHGEF38
**rs6728483**	0.022	0.007	1	42.20%	MIR3681HG
**rs17687396**	0.022	0.008	1	9.32%	
**rs2396053**	0.022	0.007	1	50.41%	
**rs4963442**	0.022	0.010	2	93.02%	
**rs7607618**	0.022	0.007	1	69.05%	ENSG00000230975
**rs1670314**	0.022	0.010	2	25.37%	TSPAN18,TP53I11
**rs6491952**	0.022	0.011	2	41.05%	
**rs16900295**	0.022	0.008	1	0.95%	
**rs1461841**	0.022	0.007	1	57.17%	ENSG00000249413

SNP	|Importance|	Std	Degree	Rank %	Gene
**rs3758126**	0.022	0.007	1	77.91%	ST18
**rs2007639**	0.022	0.009	2	45.16%	ENSG00000265174,LINC01387
**rs4673664**	0.022	0.008	1	40.88%	ERBB4
**rs205661**	0.022	0.011	2	81.33%	
**rs12533818**	0.022	0.012	2	78.21%	
**rs9880478**	0.022	0.008	1	18.36%	CFAP20DC-DT
**rs10502900**	0.022	0.010	2	96.62%	
**rs1023551**	0.022	0.008	1	61.52%	HSPA12A
**rs7829473**	0.022	0.009	1	46.06%	
**rs1576168**	0.021	0.006	1	26.46%	
**rs17506627**	0.021	0.010	3	67.35%	ENSG00000236230
**rs920980**	0.021	0.004	1	24.68%	TRIP13
**rs10899256**	0.021	0.006	1	21.46%	GUCY2EP,ENSG00000254975
**rs17618877**	0.021	0.007	1	37.57%	EXPH5
**rs2104051**	0.021	0.006	1	58.39%	FRMD6,FRMD6-AS2
**rs2292750**	0.021	0.009	5	54.63%	TUBG2
**rs11708935**	0.021	0.008	1	68.45%	
**rs160702**	0.021	0.008	1	67.49%	WRNIP1
**rs4765088**	0.021	0.007	1	51.76%	LINC02347
**rs17004824**	0.021	0.006	1	3.79%	CABIN1
**rs7364201**	0.021	0.007	1	36.58%	SYN3
**rs17149955**	0.021	0.008	1	49.72%	TMEM135
**rs6424909**	0.021	0.005	2	20.25%	RGL1
**rs2491430**	0.021	0.005	2	23.17%	RGL1
**rs11727464**	0.021	0.010	2	88.59%	GRID2

SNP	|Importance|	Std	Degree	Rank %	Gene
**rs1862856**	0.021	0.008	1	3.56%	ENSG00000259283
**rs863582**	0.021	0.007	1	85.53%	MUC4
**rs2064937**	0.021	0.009	1	78.00%	COMMD7,ENSG00000285382
**rs1537834**	0.021	0.018	3	42.52%	
**rs1333748**	0.021	0.007	2	43.84%	NALCN
**rs2375957**	0.021	0.009	1	43.84%	PUM3
**rs12863927**	0.021	0.009	2	57.10%	
**rs17029539**	0.021	0.008	1	8.51%	
**rs2643823**	0.020	0.009	1	15.55%	
**rs912594**	0.020	0.006	1	93.24%	LINC00457,LINC02343
**rs4961719**	0.020	0.007	1	78.52%	BNC2
**rs17610444**	0.020	0.006	1	26.59%	
**rs12805702**	0.020	0.009	1	46.04%	
**rs1557142**	0.020	0.011	2	32.04%	PDE7B
**rs7222667**	0.020	0.014	2	68.68%	ASIC2
**rs436463**	0.020	0.009	1	33.42%	DSEL-AS1
**rs7656598**	0.020	0.007	1	27.92%	
**rs2368045**	0.020	0.007	1	27.83%	LINC02036
**rs12833553**	0.020	0.008	1	19.04%	GABRA3
**rs5925085**	0.020	0.009	1	78.09%	
**rs7646077**	0.020	0.007	1	42.12%	LSAMP
**rs1077084**	0.020	0.007	1	3.53%	TCERG1L
**rs11644000**	0.020	0.011	2	84.14%	
**rs1469101**	0.020	0.008	1	58.62%	
**rs17803698**	0.020	0.007	1	64.54%	CLEC16A
**rs964744**	0.020	0.007	1	41.04%	

SNP	|Importance|	Std	Degree	Rank %	Gene
**rs1019738**	0.020	0.006	1	54.88%	
**rs1965528**	0.020	0.007	1	32.43%	MAGI2
**rs6575242**	0.020	0.007	1	21.62%	
**rs1288829**	0.020	0.007	1	97.88%	FOXP1,ENSG00000285708
**rs2561078**	0.020	0.007	1	44.53%	ENSG00000251391
**rs10979372**	0.020	0.013	3	85.94%	
**rs11919795**	0.020	0.008	1	4.80%	DVL3
**rs6096234**	0.019	0.008	1	92.67%	
**rs4851675**	0.019	0.007	1	87.78%	
**rs549476**	0.019	0.008	1	19.41%	NEDD4L
**rs3773300**	0.019	0.007	1	41.29%	FBLN2
**rs710394**	0.019	0.004	1	56.82%	LINC02144
**rs17618119**	0.019	0.010	2	42.90%	PTCD3
**rs9394**	0.019	0.007	1	61.44%	PKP2
**rs2268682**	0.019	0.006	1	24.76%	PRPH2
**rs1954338**	0.019	0.007	1	97.36%	
**rs10157978**	0.019	0.006	2	53.66%	
**rs11144411**	0.019	0.007	1	20.10%	
**rs12570033**	0.019	0.010	2	3.81%	STK32C
**rs1885697**	0.019	0.008	1	54.52%	TIAM2
**rs4854526**	0.019	0.007	1	31.96%	
**rs12491199**	0.019	0.006	1	11.23%	THRB,ENSG00000289130
**rs4782574**	0.019	0.009	1	48.30%	OSGIN1
**rs7320534**	0.019	0.006	2	44.44%	NALCN
**rs1333746**	0.019	0.006	2	44.44%	NALCN
**rs761857**	0.019	0.007	1	11.67%	ENSG00000286787

SNP	|Importance|	Std	Degree	Rank %	Gene
**rs9548962**	0.019	0.010	2	36.76%	
**rs12763104**	0.019	0.007	1	87.36%	
**rs17822848**	0.019	0.007	1	81.18%	ZPLD1
**rs1791523**	0.019	0.007	1	66.66%	LINC01915,ENSG00000266489
**rs1412444**	0.018	0.008	1	60.76%	LIPA
**rs2143511**	0.018	0.007	1	86.53%	PTPN1
**rs8025774**	0.018	0.009	2	4.11%	SMAD3
**rs10259381**	0.018	0.007	1	85.23%	ZNF804B
**rs7647357**	0.018	0.007	1	18.08%	PTPRG
**rs2783424**	0.018	0.007	1	56.59%	
**rs12454377**	0.018	0.008	1	65.86%	
**rs2376305**	0.018	0.008	1	59.63%	SMARCA2
**rs1034020**	0.018	0.006	3	59.14%	ENSG00000236230
**rs2236490**	0.018	0.006	1	14.17%	
**rs1442973**	0.018	0.008	1	90.85%	MAP3K20-AS1
**rs1324758**	0.018	0.006	1	66.09%	LINC00457,LINC02343
**rs17045459**	0.018	0.008	1	37.90%	
**rs4846479**	0.018	0.008	2	58.21%	TGFB2
**rs4813802**	0.018	0.011	3	51.12%	
**rs6893509**	0.018	0.008	1	97.97%	
**rs7718785**	0.018	0.006	3	72.04%	
**rs275467**	0.018	0.007	1	23.19%	
**rs7081563**	0.018	0.007	1	34.94%	ENSG00000287277
**rs7922731**	0.018	0.007	1	91.04%	LINC01163
**rs9822689**	0.018	0.010	2	4.66%	
**rs4871322**	0.018	0.008	2	36.56%	ZHX2

SNP	|Importance|	Std	Degree	Rank %	Gene
**rs852517**	0.018	0.009	1	40.49%	RNF216
**rs12013673**	0.018	0.008	2	77.53%	FOXO4
**rs1524100**	0.018	0.007	1	11.50%	
**rs11210527**	0.018	0.007	1	7.53%	HIVEP3
**rs6431233**	0.018	0.008	1	39.65%	
**rs8039531**	0.018	0.013	3	76.68%	LINC02351
**rs9109**	0.017	0.007	1	17.15%	PAK2
**rs10895090**	0.017	0.008	1	63.83%	
**rs285334**	0.017	0.014	3	29.52%	
**rs9891768**	0.017	0.008	1	54.33%	ENSG00000265542
**rs10839658**	0.017	0.011	2	32.02%	OR2D3,ENSG00000283415
**rs12919297**	0.017	0.007	1	41.36%	
**rs286437**	0.017	0.007	1	16.54%	SPEF2
**rs12758963**	0.017	0.007	1	85.00%	ENSG00000286263
**rs7990415**	0.017	0.007	1	17.66%	
**rs17154753**	0.017	0.010	2	84.21%	NXPH1
**rs17566445**	0.017	0.008	1	75.87%	ERO1B
**rs8033723**	0.017	0.007	1	67.15%	PCSK6
**rs10428352**	0.017	0.007	1	92.68%	STX18-AS1
**rs16843438**	0.017	0.009	1	77.91%	ANO7
**rs6993971**	0.017	0.009	1	87.21%	SAMD12
**rs28627320**	0.017	0.008	1	42.14%	
**rs17818937**	0.017	0.006	1	97.67%	LINC02069
**rs10156332**	0.017	0.008	1	83.58%	ENSG00000253901
**rs8024690**	0.017	0.007	1	40.78%	ADAMTS17
**rs10257697**	0.017	0.007	1	24.95%	

SNP	|Importance|	Std	Degree	Rank %	Gene
**rs2855142**	0.017	0.008	1	88.41%	TRIM21
**rs707645**	0.017	0.007	1	27.54%	ENSG00000249941
**rs11787759**	0.017	0.006	1	82.68%	
**rs1641788**	0.017	0.009	2	57.70%	NUBP1,TVP23A
**rs713333**	0.016	0.008	1	23.38%	
**rs2252324**	0.016	0.007	1	72.06%	
**rs1174656**	0.016	0.005	2	18.34%	RGL1,APOBEC4
**rs4392367**	0.016	0.010	2	43.89%	CRTAP
**rs8891**	0.016	0.008	1	47.41%	DNAJC30
**rs17143272**	0.016	0.015	2	70.53%	ABCB5
**rs10063281**	0.016	0.011	2	29.97%	KCNIP1
**rs10452035**	0.016	0.007	1	73.69%	
**rs15969**	0.016	0.007	1	13.43%	RBM14,RBM14-RBM4
**rs10877902**	0.016	0.008	1	2.03%	PPM1H
**rs11143040**	0.016	0.006	1	87.55%	C9orf85
**rs6635708**	0.016	0.007	1	16.16%	
**rs12518000**	0.016	0.006	1	56.51%	TBC1D9B
**rs10102960**	0.016	0.009	2	52.71%	CSMD1
**rs1243446**	0.016	0.008	1	81.79%	NDRG2
**rs1551929**	0.016	0.010	2	96.67%	
**rs755048**	0.016	0.007	2	64.55%	HCN1
**rs4511648**	0.016	0.007	1	14.44%	ZNF536
**rs747580**	0.016	0.009	2	78.75%	ACOX3
**rs1158541**	0.016	0.009	1	53.20%	GRIN2B
**rs13100540**	0.016	0.007	1	4.41%	

SNP	|Importance|	Std	Degree	Rank %	Gene
**rs7333705**	0.016	0.007	1	7.40%	SPATA13,ENSG00000273167
**rs1125102**	0.016	0.006	1	71.62%	CNTN4
**rs11779911**	0.016	0.008	1	90.52%	SIRLNT
**rs4763132**	0.016	0.007	1	67.40%	SRGAP1
**rs13264706**	0.016	0.009	2	44.24%	ZHX2
**rs1490343**	0.016	0.010	2	45.38%	
**rs200994**	0.015	0.007	1	52.62%	H2BC15
**rs8111234**	0.015	0.007	1	95.15%	
**rs6748459**	0.015	0.006	1	19.49%	
**rs354519**	0.015	0.015	2	19.37%	TUSC3
**rs35665**	0.015	0.008	1	35.57%	ENSG00000282863
**rs10489762**	0.015	0.005	2	15.11%	
**rs951818**	0.015	0.007	1	30.19%	CD4
**rs9289163**	0.015	0.007	1	71.56%	STXBP5L
**rs4128294**	0.015	0.009	1	69.50%	TBC1D5
**rs3791423**	0.015	0.008	1	11.74%	HDAC4,ENSG00000286307
**rs4968558**	0.015	0.010	2	84.61%	BCAS3,ENSG00000267131
**rs1022663**	0.015	0.011	2	55.67%	GALNT2
**rs293875**	0.015	0.010	1	90.09%	CSMD1
**rs6625546**	0.015	0.007	1	86.87%	EDA
**rs13185587**	0.015	0.004	2	99.79%	
**rs10494460**	0.015	0.007	1	20.35%	
**rs12852829**	0.015	0.008	2	75.89%	MED12
**rs4298088**	0.015	0.006	1	29.56%	EVC2
**rs4823984**	0.015	0.008	2	65.35%	
**rs2992753**	0.015	0.005	1	78.85%	KLHDC7A

SNP	|Importance|	Std	Degree	Rank %	Gene
**rs10839548**	0.015	0.009	1	80.10%	
**rs960633**	0.015	0.007	1	66.75%	
**rs3014771**	0.015	0.009	2	69.94%	
**rs1039797**	0.015	0.004	2	99.26%	
**rs1483401**	0.014	0.006	1	13.82%	DLG2
**rs2691809**	0.014	0.008	1	33.30%	SCIN
**rs12203454**	0.014	0.005	1	43.65%	
**rs13023050**	0.014	0.007	1	37.24%	LRP1B
**rs179428**	0.014	0.013	3	51.73%	KCNQ1
**rs6783810**	0.014	0.014	3	76.49%	CACNA1D
**rs10850475**	0.014	0.006	1	38.78%	
**rs10497454**	0.014	0.010	2	7.78%	
**rs11209405**	0.014	0.008	1	74.68%	LINC01707,LINC02791
**rs7886010**	0.014	0.007	1	68.24%	
**rs17724172**	0.014	0.007	1	31.85%	DLGAP1
**rs10152049**	0.014	0.007	1	50.68%	NRXN3
**rs6739713**	0.014	0.006	2	52.82%	
**rs2389910**	0.014	0.007	1	5.61%	RAP2A
**rs16957568**	0.014	0.007	1	67.05%	ENSG00000261285
**rs26075**	0.014	0.005	1	5.66%	RNF130
**rs6731361**	0.014	0.007	1	40.57%	
**rs4901210**	0.014	0.007	1	61.07%	
**rs10994470**	0.014	0.006	1	23.43%	MSMB
**rs2992757**	0.014	0.004	1	82.81%	
**rs13190616**	0.014	0.008	1	47.78%	LINC02163
**rs12481143**	0.014	0.008	1	59.83%	SAMHD1

SNP	|Importance|	Std	Degree	Rank %	Gene
**rs16864923**	0.013	0.009	2	44.66%	
**rs11709691**	0.013	0.007	1	58.10%	ZNF589
**rs772622**	0.013	0.007	3	20.25%	
**rs1530559**	0.013	0.010	1	93.21%	MAP3K19
**rs5749468**	0.013	0.011	1	94.22%	SYN3
**rs12676128**	0.013	0.007	1	67.52%	
**rs13225906**	0.013	0.007	1	98.66%	MDFIC
**rs8123674**	0.013	0.007	1	93.22%	
**rs4818067**	0.013	0.007	1	49.70%	B3GALT5,ENSG00000225330
**rs10906888**	0.013	0.006	1	68.71%	PRKCQ
**rs12890229**	0.013	0.008	1	26.43%	
**rs2078450**	0.013	0.007	1	49.09%	ZBTB40
**rs7728035**	0.013	0.006	1	32.28%	
**rs12913551**	0.013	0.007	1	79.18%	RAB8B
**rs2179218**	0.013	0.008	1	93.34%	PTPRT
**rs200968**	0.013	0.008	2	33.70%	H3C12
**rs2208105**	0.013	0.008	1	67.25%	LANCL3,ENSG00000250349
**rs9593136**	0.013	0.008	1	10.80%	LMO7DN,LMO7DN-IT1
**rs10104063**	0.013	0.006	1	14.69%	TUSC3
**rs2631746**	0.013	0.007	1	97.50%	TRMT44,ENSG00000205959
**rs2068701**	0.012	0.008	1	59.68%	ENSG00000288035
**rs1332290**	0.012	0.011	2	91.41%	IL33
**rs12675721**	0.012	0.006	1	37.07%	
**rs4076553**	0.012	0.007	1	89.05%	
**rs518004**	0.012	0.006	1	25.27%	

SNP	|Importance|	Std	Degree	Rank %	Gene
**rs10977362**	0.012	0.010	2	96.79%	PTPRD
**rs8096586**	0.012	0.009	2	60.22%	ENSG00000263745
**rs2512987**	0.012	0.011	2	61.29%	ENSG00000254731
**rs12125471**	0.012	0.008	1	26.70%	NEGR1,ZRANB2-DT
**rs6454367**	0.012	0.008	1	48.47%	
**rs2797129**	0.012	0.006	1	59.48%	
**rs714647**	0.012	0.006	1	13.79%	PCSK6
**rs13260745**	0.012	0.007	1	62.63%	
**rs454422**	0.012	0.007	1	93.66%	MCM8,ENSG00000286235
**rs788159**	0.012	0.006	1	70.80%	METAP1D
**rs1275528**	0.012	0.006	2	41.91%	SLC5A6
**rs12716868**	0.012	0.007	1	82.74%	WWOX,ENSG00000260511
**rs12975551**	0.012	0.007	1	25.91%	ELSPBP1
**rs11934651**	0.012	0.008	1	92.66%	
**rs1565654**	0.012	0.011	1	63.72%	TMEM132D
**rs3007733**	0.012	0.005	1	95.77%	
**rs4234618**	0.012	0.007	1	5.10%	
**rs11012390**	0.011	0.008	1	33.58%	NEBL
**rs1423363**	0.011	0.005	1	71.45%	RAB3C
**rs2546048**	0.011	0.007	1	2.20%	LINC02965,ENSG00000271109
**rs10802064**	0.011	0.008	1	66.17%	TBX15
**rs2161719**	0.011	0.008	1	4.95%	WWOX
**rs1552973**	0.011	0.007	1	59.14%	ENSG00000251095
**rs7221762**	0.011	0.005	1	23.80%	RTN4RL1
**rs10073636**	0.011	0.005	2	61.45%	HCN1

SNP	|Importance|	Std	Degree	Rank %	Gene
**rs7320590**	0.011	0.005	1	22.47%	
**rs34692025**	0.011	0.007	1	10.02%	MAGI2
**rs6683598**	0.011	0.011	3	56.59%	TGFB2
**rs7546194**	0.011	0.007	1	58.34%	ADGRL2
**rs6578908**	0.011	0.010	1	98.29%	ENSG00000254951,ENSG00000271758
**rs783304**	0.011	0.009	1	11.09%	ST3GAL3,ENSG00000284989
**rs6020972**	0.011	0.009	1	93.73%	
**rs4787348**	0.011	0.007	1	55.53%	HS3ST4
**rs4961195**	0.011	0.007	1	73.69%	RMDN1
**rs3900446**	0.011	0.008	1	75.99%	
**rs7506177**	0.011	0.007	1	32.25%	
**rs2376308**	0.011	0.012	2	73.94%	SMARCA2
**rs12195791**	0.011	0.011	2	6.28%	
**rs1274762**	0.011	0.007	1	34.53%	RAD51B
**rs3095502**	0.010	0.007	1	66.19%	
**rs1564954**	0.010	0.007	1	45.62%	KCNH7
**rs525007**	0.010	0.007	1	25.87%	
**rs2862092**	0.010	0.006	1	78.19%	
**rs10919851**	0.010	0.007	1	83.60%	
**rs6730157**	0.010	0.006	2	82.20%	RAB3GAP1,ZRANB3
**rs1473527**	0.010	0.015	4	68.09%	
**rs902966**	0.010	0.007	1	0.54%	
**rs6977284**	0.010	0.007	1	70.88%	ENSG00000226965
**rs10842635**	0.009	0.008	1	61.53%	
**rs2476934**	0.009	0.006	1	95.11%	SUSD1

SNP	|Importance|	Std	Degree	Rank %	Gene
**rs795085**	0.009	0.007	1	97.66%	
**rs12465802**	0.009	0.006	2	74.70%	R3HDM1
**rs825839**	0.009	0.007	1	41.58%	ZFHX3,LINC01568
**rs7205**	0.009	0.007	1	56.90%	SLC25A6
**rs10958390**	0.009	0.006	1	32.73%	
**rs3891910**	0.009	0.007	1	40.19%	
**rs11739923**	0.009	0.005	1	53.42%	
**rs2194193**	0.008	0.007	1	78.47%	
**rs185763**	0.008	0.014	3	24.01%	TENM2
**rs4844285**	0.008	0.009	2	79.35%	NLGN3
**rs4788808**	0.008	0.007	1	85.47%	
**rs3922385**	0.008	0.006	1	12.80%	ENSG00000287684
**rs4689767**	0.008	0.012	1	38.35%	SORCS2
**rs236175**	0.008	0.008	1	79.76%	TRMT6
**rs6735844**	0.008	0.007	1	14.39%	MYO3B
**rs13020966**	0.008	0.008	1	87.47%	
**rs2423366**	0.008	0.008	1	72.64%	PLCB1
**rs11929934**	0.008	0.008	1	13.29%	SCFD2
**rs12476**	0.008	0.007	1	7.94%	AAGAB
**rs951747**	0.008	0.006	1	55.90%	OR51B5,HBG2,HBE1,ENSG00000239920
**rs13255611**	0.007	0.008	1	88.78%	
**rs9961643**	0.007	0.010	1	86.76%	MYO5B
**rs4758468**	0.007	0.006	1	16.55%	OSBPL5
**rs11940750**	0.007	0.009	1	12.96%	TENM3
**rs1517002**	0.007	0.006	3	57.91%	RETREG3
**rs11649877**	0.007	0.006	3	57.91%	

SNP	|Importance|	Std	Degree	Rank %	Gene
**rs1561535**	0.007	0.004	1	26.98%	
**rs10823610**	0.007	0.008	1	22.83%	ADAMTS14
**rs17061088**	0.007	0.013	1	62.67%	ENSG00000254186
**rs1456079**	0.007	0.006	1	31.66%	STAC
**rs12048672**	0.007	0.006	1	50.59%	
**rs2543139**	0.006	0.014	2	12.62%	TUSC3
**rs951157**	0.006	0.007	1	62.02%	OPCML
**rs12148908**	0.006	0.007	1	70.09%	GABRG3
**rs7223643**	0.006	0.010	2	33.69%	BPTF
**rs3011415**	0.006	0.006	1	49.58%	LINC02930
**rs11796153**	0.006	0.006	1	79.01%	MED12
**rs13157385**	0.006	0.008	1	29.55%	RAB3C,ENSG00000248475
**rs5997629**	0.006	0.007	1	48.50%	RNF215
**rs6922018**	0.006	0.007	1	51.64%	BLOC1S5-TXNDC5
**rs1024091**	0.006	0.003	1	63.52%	
**rs28469650**	0.006	0.006	1	74.90%	PCSK6
**rs979607**	0.006	0.012	4	75.87%	
**rs4793244**	0.006	0.005	2	57.99%	
**rs16928064**	0.006	0.009	2	50.49%	LINC02842
**rs11126931**	0.006	0.006	1	19.23%	
**rs2409086**	0.005	0.011	2	18.83%	ENSG00000254367
**rs17580421**	0.005	0.008	1	82.56%	
**rs2311074**	0.005	0.009	2	85.97%	
**rs9352063**	0.005	0.007	1	9.20%	ENSG00000223786
**rs2833866**	0.005	0.008	1	78.49%	TCP10L,CFAP298-TCP10L

SNP	|Importance|	Std	Degree	Rank %	Gene
**rs2835270**	0.005	0.007	1	50.81%	SETD4,CBR1-AS1
**rs7571877**	0.005	0.008	1	6.93%	
**rs10265823**	0.005	0.008	1	7.91%	ELMO1
**rs7373930**	0.005	0.008	1	54.67%	XYLB
**rs10927866**	0.005	0.008	1	90.87%	SPEN
**rs6574751**	0.005	0.008	1	42.71%	
**rs10940346**	0.004	0.005	1	84.24%	
